# Extracellular matrix-degrading STING nanoagonists for mild NIR-II photothermal-augmented chemodynamic-immunotherapy

**DOI:** 10.1186/s12951-021-01226-3

**Published:** 2022-01-06

**Authors:** Meixiao Zhan, Xiangrong Yu, Wei Zhao, Yongjun Peng, Shaojun Peng, Jingchao Li, Ligong Lu

**Affiliations:** 1 Zhuhai Institute of Translational Medicine, Guangdong Provincial Key Laboratory of Tumor Interventional Diagnosis and Treatment, Zhuhai People’s Hospital (Zhuhai Hospital Affiliated with Jinan University), Zhuhai, 519000 Guangdong China; 2 Shanghai Engineering Research Center of Nano-Biomaterials and Regenerative Medicine, College of Chemistry, Chemical Engineering and Biotechnology, Donghua University, Shanghai, 201620 China

**Keywords:** STING pathway, Immunotherapy, Fenton reaction, Photoactivation, Nanoagonist

## Abstract

Regulation of stimulator of interferon genes (STING) pathway using agonists can boost antitumor immunity for cancer treatment, while the rapid plasma clearance, limited membrane permeability, and inefficient cytosolic transport of STING agonists greatly compromise their therapeutic efficacy. In this study, we describe an extracellular matrix (ECM)-degrading nanoagonist (dNAc) with second near-infrared (NIR-II) light controlled activation of intracellular STING pathway for mild photothermal-augmented chemodynamic-immunotherapy of breast cancer. The dNAc consists of a thermal-responsive liposome inside loading with ferrous sulfide (FeS_2_) nanoparticles as both NIR-II photothermal converters and Fenton catalysts, 2′3′-cyclic guanosine monophosphate-adenosine monophosphate (cGAMP) as the STING agonist, and an ECM-degrading enzyme (bromelain) on the liposome surface. Mild heat generated by dNAc upon NIR-II photoirradiation improves Fenton reaction efficacy to kill tumor cells and cause immunogenic cell death (ICD). Meanwhile, the generated heat triggers a controlled release of cGAMP from thermal-responsive liposomes to active STING pathway. The mild photothermal activation of STING pathway combined with ICD promotes anti-tumor immune responses, which leads to improved infiltration of effector T cells into tumor tissues after bromelain-mediated ECM degradation. As a result, after treatment with dNAc upon NIR-II photoactivation, both primary and distant tumors in a murine mouse model are inhibited and the liver and lung metastasis are effectively suppressed. This work presents a photoactivatable system for STING pathway and combinational immunotherapy with improved therapeutic outcome.

**Supplementary Information:**

The online version contains supplementary material available at 10.1186/s12951-021-01226-3.

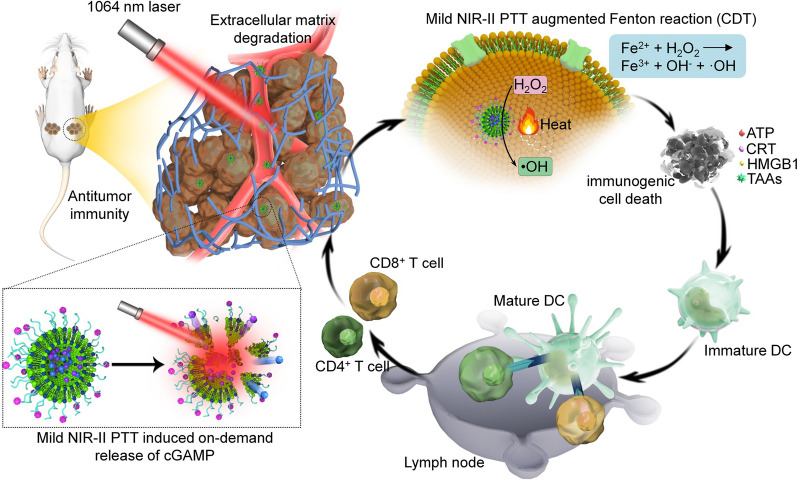

## Introduction

Immunotherapy can eliminate local and disseminated metastatic tumors through stimulating the host’s antitumor immune responses, and establish an effective immune memory to avoid tumor recurrence [1–3]. Immune checkpoint blockade therapy that uses immune checkpoint inhibitors to reverse inhibitory pathways between immune effector cells and tumor cells has been approved for the treatment of several types of cancer [4–6]. However, this therapeutic strategy is often inoperative for clinic patients [7]. Only a small fraction of patients can be cured by immune checkpoint inhibitors due to their immunogenic ‘hot’ tumor microenvironment infiltrated with antigen-specific T cells [8–10], while a large subset of patients have nonimmunogenic ‘cold’ tumors with poor T-cell infiltration and thus show low response ratios to immune checkpoint inhibitors [11]. The combination of multiple immune checkpoint blockers can increase patients’ responses, while this potentially results in severe immune-related adverse events [12–14].

As the cytosolic pattern recognition receptor for cyclic dinucleotides (CDNs), stimulator of interferon genes (STING) shows a critical role in cancer immune surveillance [15, 16]. Activation of STING can accelerate maturation of dendritic cells (DCs), antigen presentation, and priming of immune T cells [17, 18]. CDNs that can bind with STING dimers have been used as therapeutic agonists to trigger antitumor immunity for cancer treatment [19–21]. However, the therapeutic efficacy of CDNs is often limited because of their rapid plasma clearance, poor cell membrane permeability, and inefficient transport into cytosol [22, 23].

As the progress of nanotechnology, different nanoparticles or nanocomposites have been developed [24–26]. These nanomaterials with different properties can be used for imaging [27–29], sensing [30–33], controlled drug delivery [34] and cancer therapy [35]. Recently, drug delivery nanosystems including liposomes [36, 37], polymersomes [38], polymer nanoparticles [39], inorganic nanoparticles [40], and hydrogels [41] have been constructed to improve the bioavailability of CDNs, while their controlled release and accumulation at targeting regions (such as tumors) should be improved to reduce immune-related adverse events and increase therapeutic efficacies.

Photothermal therapy (PTT) that converts external light into heat for localized thermal damages by using photothermal agents has been employed as a method for treatment of solid tumors because of minimal invasiveness and simple operation of light [42–44]. Near-infrared (NIR) light (NIR-I, 700–950 nm) is an exogenous light source for PTT, which however has poor tissue penetrating capability and limited skin maximum permissible exposure [45–47]. In contrast, the second NIR light (NIR-II, 1000–1300 nm) with improved biological penetration and maximum permissive energy has allowed for more effective NIR-II PTT [48–50]. Apart from direct eradication of tumors, PTT has been demonstrated to enhance the efficacies of other therapeutic modality, such as chemotherapy [51], gene therapy [52], chemodynamic therapy (CDT) [53], photodynamic therapy [54], thermodynamic therapy [55], and immunotherapy [56]. In addition, PTT-mediated thermal effect can achieve on-demand release of cargos from temperature-responsive nanoparticles for high-precision combinational therapy [57–59]. Recently, febrile temperature during PTT is reported to induce immune responses through various mechanisms, while which will be dampened if tumors are heated to > 45 °C [2]. By contrast, mild PTT with a relatively low temperature at ~ 45 °C can produce favorable tumor microenvironment for immunological responses, acting as an assistance for tumor treatment [60]. In addition, high temperature that uses to ablate tumors potentially leads to damages of nearby normal tissues because of nonspecific heating and heat diffusion [61, 62]. Therefore, cancer treatment via PTT with a mild heating will be important for effective and safe therapy. However, mild PTT mediated activation of STING pathway and combinational immunotherapy has not been reported.

**Fig. 1:**
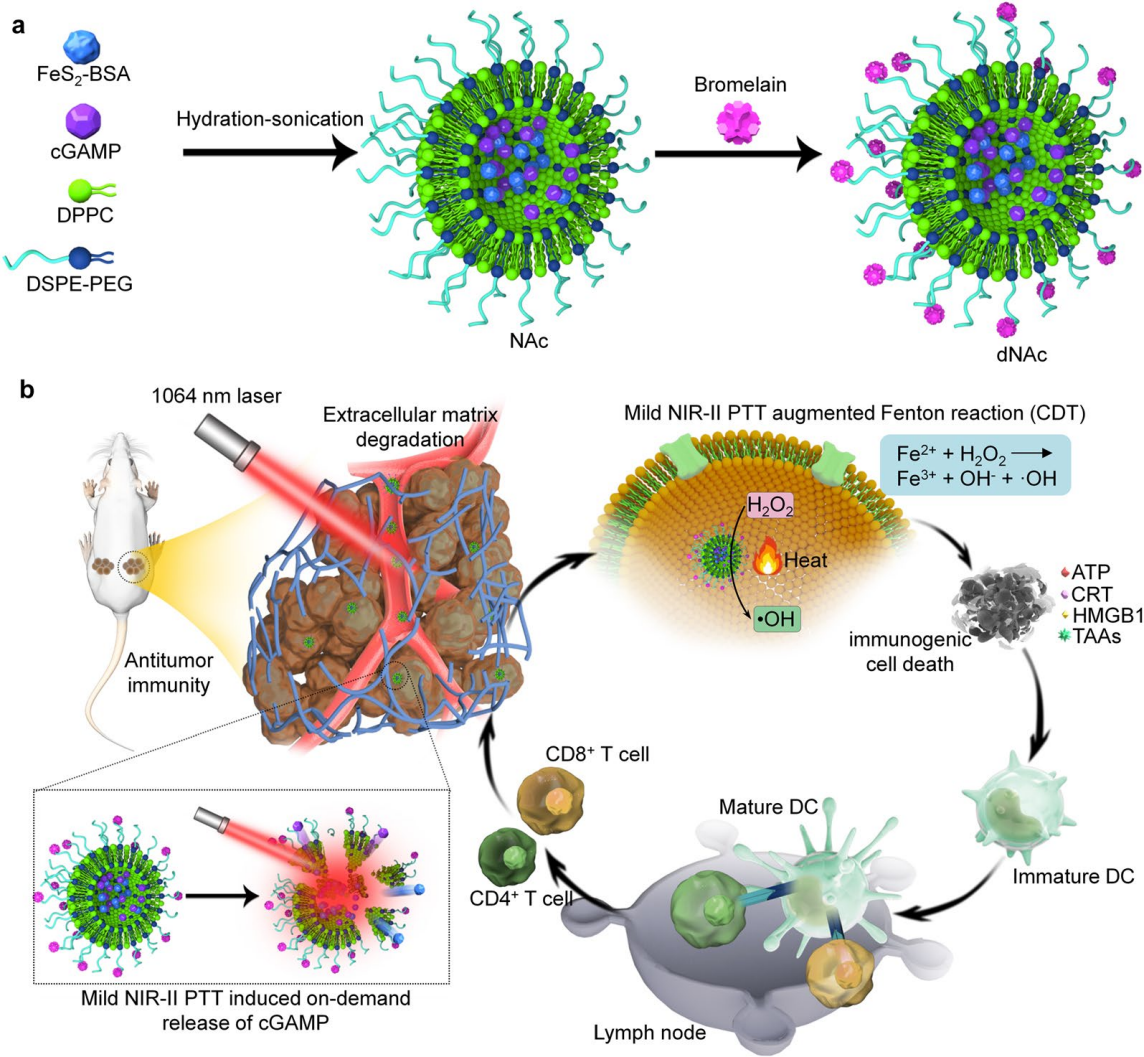
ECM-degrading photothermal nanoagonist (dNAc)-mediated mild NIR-II PTT-augmented CDT-immunotherapy. **a** Schematic illustration of synthesis route of dNAc via hydration-sonication and surface modification. **b** Schematic illustration of NIR-II photoactivation of dNAc for mild photothermal effect-augmented CDT-immunotherapy

In this study, we report the synthesis of an extracellular matrix (ECM)-degrading nanoagonist (dNAc) with NIR-II light controlled activation of intracellular STING pathway for mild PTT-augmented CDT-immunotherapy. The dNAc is constructed via loading 2′3′-cyclic guanosine monophosphate-adenosine monophosphate (cGAMP) as the STING agonist and NIR-II-absorbing ferrous sulfide (FeS_2_) nanoparticles into thermal-responsive liposomes, followed by surface modification with an ECM-degrading enzyme, bromelain (Fig. 1a). FeS_2_ nanoparticles can be used as not only NIR-II photothermal converters for PTT, but also Fenton catalysts for CDT. Fe^2+^ within FeS_2_ nanoparticles could react with hydrogen peroxide (H_2_O_2_) in tumor microenvironment to generate highly toxic hydroxyl radicals (·OH) for CDT [63]. Upon NIR-II laser treatment, FeS_2_-enabled generation of mild heat in tumor sites, which leads to improved Fenton reaction efficacy of FeS_2_ to kill tumor cells and cause immunogenic cell death (ICD). In addition, such a mild PTT effect allows for controlled release of cGAMP through melting thermal-responsive liposomes for activation of STING pathway (Fig. 1b). Such a procedural action can facilitate maturation of DCs, activation of effector T cells, and secretion of immune-relevant cytokines. Additionally, the surface modified bromelain degrades ECM in tumor microenvironment, which increases the intratumor infiltration of effector T cells for augmented antitumor immunity. As a result, dNAc-mediated mild photothermal effect-augmented CDT and immunotherapy obviously inhibits the growths of primary and distant 4T1 tumors, and almost completely eliminates lung and liver metastasis using subcutaneous mouse tumor models.

## Materials and methods

### Materials

Sulfur powder, bovine serum albumin (BSA), 1-dodecanethiol, hydrogen peroxide (H_2_O_2_), iron dichloride, and oleylamine were purchased from Aladdin (Shanghai, China). cGAMP was purchased from MedChemExpress (USA). Penicillin–streptomycin, fetal bovine serum (FBS), and Dulbecco’s modified Eagle’s medium (DMEM) are purchased from Gibco (USA). Cell counting kit-8 (CCK-8) and 2′,7′-dichlorodihydrofluorescein diacetate (H_2_DCFDA) were purchased from KeyGen Biotech. Co., Ltd (Nanjing, China). Other materials without notes were purchased from Sigma-Aldrich (USA). All antibodies used in staining and flow cytometry assay were purchased from Biolegend (USA) or Abcam (USA).

### Characterization

Thermogravimetric analysis (TGA) was conducted using a TG 209 F1 thermogravimetric analyzer (NETZSCH Instruments Co., Ltd., Germany). A Zetasizer Nano ZS analyzer (Malvern, UK) was used to measure hydrodynamic sizes and zeta potentials of nanoparticles. A Tecnai G2 transmission electron microscope (TEM, USA) was used to capture TEM images of nanoparticles. UV–vis-NIR spectrum was recorded using a Lamba 750 spectrophotometer (Perkin Elmer, Boston, MA). Photothermal effect evaluation was conducted using a NIR-II laser (1064 nm, 1.0 W/cm^2^). A thermal infrared camera was used to record thermal images under NIR-II laser irradiation. An inductively coupled plasma optical emission spectroscopy (ICP-OES) was employed to quantify concentrations of Fe ions. A high-performance liquid chromatography (HPLC, Agilent 1260) system was used to detect the concentrations of cGAMP.

### Synthesis of BSA-coated FeS_2_ nanoparticles

FeS_2_ nanoparticles were synthesized via a facile solvothermal reaction according to the protocols reported in a previous work [64]. Briefly, iron (II) chloride (1.0 mmol) and sulfur powder (6.0 mmol) dissolved in oleylamine was mixed with 1-dodecanethiol (5 mL) under magnetic stirring for 1 h. The formed mixture solution was moved into an autoclave and then heated at 180 °C. After 18 h reaction, the mixture was cooled down to room temperature, and the black products were purified via 4 cycles of centrifugation (8000 rpm, 8 min) and washed with hexane. After being further washed with ethyl alcohol, the products were dispersed in absolute alcohol for further use. To synthesize BSA-coated FeS_2_ nanoparticles (BSA-FeS_2_), FeS_2_ nanoparticles (1 mg) were dispersed into 5 mL BSA solution (1 mg/mL) under sonication, and the resulting solution was stirred for 1 day. The obtained products were purified by centrifugation (6000 rpm, 9 min) and water washed to remove free BSA. The formed BSA-FeS_2_ nanoparticles were dispersed in water for further use.

### Synthesis of nanoagonists

A mixture was prepared by dissolving 1,2-dipalmitoyl-sn-glycero-3-phosphocholine (DPPC, 25 mg), 1,2-distearoyl-sn-glycero-3-phosphoethanolamine-N-[methoxy(polyethylene glycol)] (DSPE-PEG, 4 mg), and DSPE-PEG-NHS (1 mg) in 10 mL chloroform. A thin film was then formed via evaporation of chloroform. Aqueous solution of BSA-FeS_2_ (0.5 mg Fe) and cGAMP (0.5 mg) were mixed with the thin film. The formed mixture was hydrated at 60 °C under stirring for 30 min, followed by sonication for another 60 min in ice bath. The products were purified by centrifugation (6000 rpm, 9 min), and water washed to remove free raw materials to obtain FeS_2_ and cGAMP loaded liposomes (NAc). To synthesize dNAc, 2.5 mg NAc dispersed in 1 × PBS (phosphate buffered saline) were mixed with bromelain and the reaction was continued for 1 day. The products were purified by centrifugation (6000 rpm, 9 min) and water washed to remove free bromelain and then dNAc was obtained. FeS_2_ loaded liposomes (NA_0_) were synthesized through a similar route and used as a control.

### Hemolysis assay

Fresh blood collected from 4 to 6-week female BALB/c mice was centrifuged (1000 rpm, 10 min) to remove supernatant and then washed with 1 × PBS to obtain erythrocytes. Erythrocytes were then incubated with 1 × PBS, 1% Triton X-100 solution or PBS solutions containing NA_0_, NAc or dNAc at different Fe concentrations at room temperature for 2 h. After that, erythrocytes were precipitated via centrifugation (10,000 rpm, 1 min) to collect supernatants. To calculate hemolysis percentages, a microplate reader was used to record the optical density (O.D.) values of supernatants at 540 nm.

### Evaluation of NIR-II photothermal effect

To evaluate the in vitro photothermal performance of nanoagonists, 200 μL solutions containing NA_0_, NAc and dNAc at a Fe concentration of 50 μg/mL were treated by NIR-II laser (1 W/cm^2^) for different time. A thermal camera was used to record the solution temperature under laser irradiation every 30 s. To investigate the photothermal stability, the laser was turned on for 6 min, turned off for another 6 min and repeated for five cycles. To evaluate the tissue penetration depth of 1064 nm laser, dNAc solutions covered with chicken breast tissue with different thicknesses (0, 1, 3, 5, and 7 mm) were treated by NIR-II laser (1 W/cm^2^) for 6 min and the temperature changes were recorded.

### Evaluation of Fenton reaction efficacy

NA_0_, NAc and dNAc dispersed in 0.5 mL water (Fe concentration = 50 μg/mL) was added into 1 mL solution of 3,3′,5,5′-tetramethylbenzidine dihydrochloride (TMB, 0.12 mg/mL) and H_2_O_2_ (100 µM). The generation of ·OH was confirmed by measuring the absorbance of TMB using a Lamba 750 spectrophotometer. To evaluate the mild photothermal-amplified Fenton reaction, nanoagonists (Fe concentration = 50 μg/mL) was added into 1 mL solution of TMB (0.12 mg/mL) and H_2_O_2_ (100 µM), and the resulting solution was treated by NIR-II laser (1 W/cm^2^) in a discontinuous manner for 6 min. A thermal camera was used to monitor the temperatures of solutions, and the maximum temperature was maintained at ~ 45 °C. The absorbance of TMB was measured using a Lamba 750 spectrophotometer.

### In vitro assay of cGAMP release

The dNAc dispersed in 1 × PBS solution (Fe concentration = 50 μg/mL) was treated by NIR-II laser (1 W/cm^2^) in a discontinuous manner for different time. A thermal camera was used to monitor the temperatures of solutions, and the maximum temperature was maintained at ~ 45 °C. After photoirradiation, the solution was filtrated through filters and analyzed using HPLC to quantify the release of cGAMP.

### In vitro evaluation of cytocompatibility

To evaluate cytocompatibility of nanoagonists, 4T1 cancer cells were incubated with NA_0_, NAc or dNAc at different final Fe concentrations for 24 h. After being carefully washed with PBS solution, the cells were incubated with CCK-8 in DMEM cell culture medium for 4 h. To calculate cell viability, a microplate reader was used to record the absorbance of each well.

### In vitro CDT efficacy evaluation

4T1 cancer cells were incubated with NA_0_, NAc or dNAc (Fe concentration = 50 μg/mL) in the presence of H_2_O_2_ (100 μM) for 1 day, and then the cells were treated by NIR-II laser (1 W/cm^2^) in a discontinuous manner for 6 min. Temperature of cell culture medium was monitored and the maximum temperature was maintained at ~ 45 °C. The cells were cultured in DMEM cell culture medium for another 12 h, and then cultured in fresh DMEM cell culture medium containing CCK-8 for 2 h. To evaluate CDT efficacy, cell viabilities of 4T1 cancer cells after different treatments were calculate by measuring the absorbance of cell culture medium using a microplate reader.

### Evaluation of intracellular ·OH production

4T1 cancer cells were cultured in DMEM cell culture medium containing H_2_DCFDA (10 μM), NA_0_, NAc or dNAc (50 μg/mL) in the absence or presence of H_2_O_2_ (100 μM) for 6 h, and then treated by NIR-II laser (1 W/cm^2^) in a discontinuous manner for 6 min. The maximum temperature was maintained at ~ 45 °C during laser irradiation. To detect the production of ·OH, a fluorescence microscope was used to capture the fluorescence images.

### In vitro evaluation of ICD

4T1 cancer cells were incubated with NA_0_, NAc or dNAc (50 μg/mL) in the presence of H_2_O_2_ (100 μM) for 6 h, and then treated by NIR-II laser (1 W/cm^2^) in a discontinuous manner for 6 min. After further culture of cells for another 12 h, the supernatant was collected to measure adenosine triphosphate (ATP) contents using ATP determination kit. The high-mobility group box 1 (HMGB1) enzyme-linked immunosorbent assay (ELISA) kit was used to measure the extracellular release of HMGB1 in cell supernatant. Western blot (WB) assay was used to confirmed the expression of calreticulin (CRT).

### Establishment of tumor models

BALB/c mice (female, 4–6 weeks) were purchased from Institute of Laboratory Animal Science of Jinan University. All animal experiments were conducted according to the procedures permitted by the Institutional Anima Care and Treatment Committee of Jinan University (Guangzhou, China)*.* To establish 4T1 tumor-bearing BALB/c mouse models, 4T1 cancer cells (2 × 10^6^ cells/mouse) were subcutaneously implanted into right flank of mice. To establish bilateral mouse tumor models, 4T1 cancer cells (2 × 10^6^ cells/mouse) were locally implanted into subcutaneous tissues of right flank and a same amount of 4T1 cancer cells were then locally injected into subcutaneous tissues of left flank of the mice after 7 days of primary tumor implantation. When the volumes of primary tumors were estimated to be ~ 100 mm^3^, 4T1 tumor-bearing mice were used for animal experiments.

### Evaluation of tumor accumulation of nanoagonists

To evaluate tumor accumulation of nanoagonists, PBS solution (0.2 mL) of NA_0_, NAc or dNAc at the Fe concentration of 300 μg/mL was intravenously injected into tumor-bearing BALB/c mice. After injection for 2, 6, and 12 h, the mice were euthanized, and tumors were extracted and weighed. After digestion of tumor tissues in aqua regia solution for 72 h, ICP-OES was used to measure the Fe contents. In addition, the tumors of mice after intravenous injection of PBS, NA_0_, NAc and dNAc at different post-injection timepoints were collected for Prussian blue staining.

### In vivo mild NIR-II PTT of tumors

4T1 tumor-bearing BALB/c mice were systemically administrated with 0.2 mL saline, saline solution of NA_0_, NAc or dNAc (Fe concentration = 300 μg/mL). At 6 h post-injection timepoint, the tumors were treated by NIR-II laser (1 W/cm^2^) in a controlled manner for 10 min. A thermal camera was used to monitor the temperatures of tumor sites. The maximum temperature was maintained at ~ 45 °C for mild NIR-II PTT.

### In vivo evaluation of ICD biomarkers in tumor tissues

4T1 tumor-bearing BALB/c mice were systemically administrated with 0.2 mL saline, saline solution of NA_0_, NAc or dNAc (Fe concentration = 300 μg/mL). At 6 h post-injection timepoint, the tumors were treated by NIR-II laser (1 W/cm^2^) in a controlled manner for 10 min. The maximum temperature of tumors was maintained at ~ 45 °C for mild NIR-II PTT. To evaluate ICD, the mice after treatments were euthanized, and the tumors were extracted, weighed, and then homogenized. The obtained suspension solution was centrifuged to collect supernatant, following by measurement of ATP levels using ATP determination kit according to the manufacture protocols. To evaluate the expressions of CRT and HMGB1, the collected tumor tissues were cut into sections and used for immunofluorescence staining. A fluorescence microscope was used to capture the fluorescence images of stained tumor sections.

### In vivo evaluation of antitumor efficacy

Bilateral 4T1 tumor-bearing BALB/c mice were systemically administrated with 0.2 mL saline, saline solution of NA_0_, NAc or dNAc (Fe concentration = 300 μg/mL). At 6 h post-injection timepoint, the primary tumors were treated by NIR-II laser (1 W/cm^2^) in a controlled manner for 10 min. The maximum temperature of tumor sites was maintained at ~ 45 °C. The sizes of primary and distant tumors from mice after different treatments were measured every 3 days for 21 days. Tumor volume was calculated as follows: V = L × W^2^/2 (the L and W represent the length (mm) and width (mm) of tumors, respectively). Relative tumor volume was calculated as V/V_0_ (V_0_ was the initial tumor volume). After 14 days of treatments, the mice in each group were euthanized, and both the primary and distant tumors were extracted for terminal deoxynucleotidyl transferase-mediated dUTP nick-end labeling (TUNEL), hematoxylin and eosin (H&E) and Ki67 staining according to the manufacture protocols. The staining images were captured using an optical microscope or a fluorescence microscope.

### In vivo evaluation of anti-metastasis efficacy

Bilateral 4T1 tumor-bearing BALB/c mice were treated according to above-described processes. At day 21, the mice in each group were euthanized, and livers and lungs were extracted. To evaluate anti-metastasis efficacy, the collected livers and lungs were assessed by H&E staining. The collected lungs were washed with PBS for three time to count the metastatic tumor nodule numbers.

### Mechanism of antitumor efficacy

After 7 or 14 days of treatment as above described, tumor draining lymph nodes (TDLNs), primary and distant tumors were extracted from mice. The TDLNs were homogenized in PBS solution to obtain single cell suspension. Then the single cells were incubated with anti-CD16 antibody, followed by staining with fluorophore labeled anti-CD11c, anti-CD45, anti-CD86 and anti-CD80 antibodies. The collected tumor tissues were treated by buffer solution of DNase I (100 μg/mL), type IV collagenase (100 μg/mL), and type I collagenase (1 mg/mL). To obtain single cell suspension, the cell suspensions were filtered using cell strainers. After treatments with lymphocyte separation medium to collect lymphocytes, the cells were treated with anti-CD16 antibody, and then stained with fluorophore labeled anti-CD3, anti-CD45, anti-CD8 and anti-CD4 antibodies. The stained DCs and T cells were analyzed using a CytoFLEX flow cytometer. Bilateral 4T1 tumor-bearing BALB/c mice were treated as described above. At day 14, primary and distant tumors were extracted from mice. The collected tumors were cut into sections for immunofluorescence staining with fluorescein isothiocyanate (FITC) labeled anti-CD4 and anti-CD8 antibody, respectively. After further staining of cell nucleus with 4′,6-diamidino-2-phenylindole (DAPI), fluorescence images of staining tumor sections were captured using the LSM800 confocal laser scanning microscope. After different treatments for 6 days, fresh blood samples were collected form bilateral 4T1 tumor-bearing BALB/c mice, and then centrifuged to obtain serum. ELISA assay kits were used to measure the levels of tumor necrosis factor-α (TNF-α), interferon-γ (IFN-γ) and interleukin-6 (IL-6) in serum according to the manufacture protocols.

### In vivo biosafety evaluation

4T1 tumor-bearing BALB/c mice were systemically administrated with 0.2 mL saline solution of BSA-FeS_2_ nanoparticles (Fe concentration = 300 μg/mL) and the contents of Fe in different tissues after injection was measured using ICP-OES. In vivo biosafety was evaluated by measuring the body weights of tumor-bearing mice after different treatments. Major organs including heart, liver, spleen, lung, and kidney were extracted from the control, BSA-FeS_2_-, and dNAc-injected mice with or without NIR-II laser treatment. H&E staining of these tissues were performed to evaluate histological morphologies. In addition, blood routine and biochemical testing of blood samples from control and different treated mice were performed.

### Statistical analysis

All experiments were duplicated for at least three times. Significant difference (* for p < 0.05, ** for p < 0.01, and *** for p < 0.001) was calculated using one-way ANOVA statistical analysis.

## Results and discussion

### Nanoagonist synthesis and characterization

The construction of dNAc consisted of the following three steps: synthesis of BSA-coated FeS_2_ nanoparticles, preparation of thermal-responsive liposomes loading with FeS_2_ and cGAMP, and surface modification of ECM-degrading enzyme. FeS_2_ nanoparticles were synthesized according to a previous study [64], and then coated with BSA to form BSA-FeS_2_. TGA showed that the weight loss of BSA-FeS_2_ nanoparticles was obvious compared to that of FeS_2_ nanoparticles (Additional file 1: Fig. S1), suggesting the successful coating of BSA onto the surface of FeS_2_ nanoparticles. The percentage of BSA coating in nanoparticles was calculated to be 44.5%. Hydration-sonication process was conducted to construct thermal-responsive liposomes using BSA-FeS_2_, cGAMP, DPPC, DSPE-PEG, and DSPE-PEG-NHS at the feeding mass ratio of 0.5:0.5:25:4:1. Further surface modification of bromelain resulted in the formation of dNAc. FeS_2_ and cGAMP loaded liposomes (termed as NAc) and FeS_2_ loaded liposomes (termed as NA_0_) without bromelain modification were synthesized via the similar processes and used as control counterparts, respectively. TGA was used to confirm the synthesis of different nanoagonists. The weight loss was calculated to be 67.3%, 68.7% and 79.3% for NA_0_, NAc and dNAc, respectively, suggesting the loading of cGAMP and surface modification of bromelain (Additional file 1: Fig. S1). The percentage of major components in dNAc (FeS_2_, cGAMP and bromelain) was calculated to be 20.7%, 1.4% and 10.6%, respectively.

**Fig. 2:**
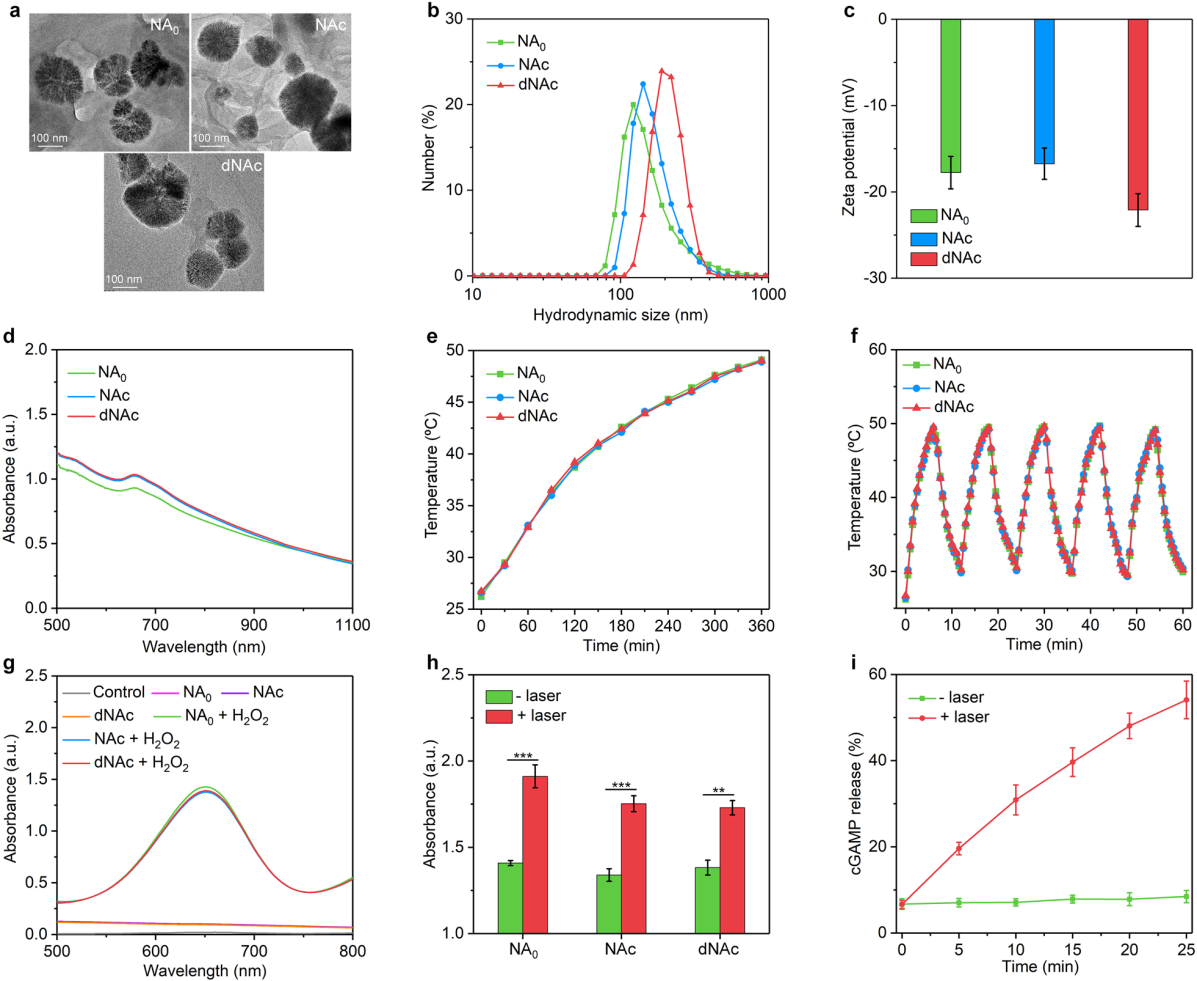
Nanoagonist characterization. **a** TEM images of NA_0_, NAc and dNAc. **b** Hydrodynamic sizes of NA_0_, NAc and dNAc measured by DLS. **c** The zeta potential of NA_0_, NAc and dNAc dispersed in water. **d** UV–Vis-NIR spectra of solution containing NA_0_, NAc and dNAc. **e** Temperature curves of NA_0_, NAc and dNAc after treatment of NIR-II laser (1 W/cm^2^) for different time. **f** Photothermal stability evaluation of NA_0_, NAc and dNAc after five cycles of laser on/off. **g** Analysis of ·OH generation via nanoagonists mediated Fenton reaction using TMB as indicator. **h** Relative ·OH generation of nanoagonists with or without NIR-II laser treatment (1 W/cm^2^) for 6 min. **i** Release of cGAMP from dNAc after NIR-II laser treatment (1 W/cm^2^) for different time

TEM images showed that NA_0_, NAc and dNAc possessed a similar morphology and dimension distribution (Fig. 2a). The hydrodynamic sizes for NA_0_, NAc and dNAc measured by dynamic light scattering (DLS) were similar within the range from 163.0 to 212.4 nm (Fig. 2b). The surface potentials were measured to be − 17.8, − 16.7 and − 22.1 mV for NA_0_, NAc and dNAc, respectively (Fig. 2c). The absorption of these nanoagonists in the NIR range was almost consistent, which indicated the loading of cGAMP and surface modification of bromelain did not affect the optical property (Fig. 2d). After storage of nanoagonists for 2 weeks, nearly no precipitates were observed, confirming their long-term good colloidal stability (Additional file 1: Fig. S2). Hemolysis assay indicated that these three types of nanoagonists had good hemocompatibility to ensure their hematologic safety for in vivo biological applications (Additional file 1: Fig. S3).

The photothermal properties and Fenton reaction efficacy of nanoagonists and mild NIR-II PTT triggered cGAMP release were investigated. Under NIR-II laser irradiation (1 W/cm^2^), the solution of NA_0_, NAc and dNAc showed gradual temperature increase in a similar manner and the temperature reached around 49.0 °C after laser irradiation for 6 min (Fig. 2e and Additional file 1: Fig. S4). The photothermal conversion efficiencies of nanoagonists were calculated to be 29.7%, which was similar as that of red blood cell membrane coated FeS_2_ nanoparticles (30.2%) [64], while higher relative to that of gold nanostars (13.0%) [65], pyroelectric nanoparticles (20.3%) [66], bismuth-based nanoparticles (24.4%) [67], and 2D MXenes nanoparticles (28.6%) [68] at 1064 nm. In addition, all these nanoagonists demonstrated excellent photothermal stability as their photothermal temperature curves remained almost unchanged after repeated laser on/off (Fig. 2f). To verify the deep penetration depth of 1064 nm laser, dNAc solutions covered with chicken breast tissue with the thickness of 0, 1, 3, 5, and 7 mm were irradiated by 1064 nm laser. The temperature gradually increased under laser irradiation for all groups (Additional file 1: Fig. S5), while the maximum temperature reduced as the increase of tissue thickness. At the tissue thickness of 5 mm, the temperature increase was measured to be 19.1 °C, which was above the required temperature rise (13 °C) for PTT [69]. Therefore, the tissue penetration depth of 1064 nm laser for PTT was 5 mm, which was consistent with that in a previous study [69].

The generation of hydroxyl radical (·OH) by FeS_2_-mediated Fenton reaction was evaluated with TMB as indicator. The characteristic absorption peak at 650 nm was observed for nanoagonists in the presence of H_2_O_2_, while which was not observed for those without addition of H_2_O_2_ (Fig. 2g), suggesting generation of ·OH via Fenton reaction for CDT. Furthermore, the relative ·OH generation for these nanoagonists exhibited 1.3 ~ 1.4-fold increase after laser irradiation compared to those without laser irradiation (Fig. 2h), suggesting that the Fenton reaction effect of nanoagonists was accelerated by NIR-II PTT mediated mild temperature rise. The thermal-responsive liposome shell was reported to be melted at the phase-transition temperature of about 42 °C [70], mild NIR-II PTT triggered on-demand release of cGAMP was then investigated. Upon treatment with NIR-II laser (1 W/cm^2^), release of cGAMP from dNAc was observed in an irradiating time dependent manner, while nearly no cGAMP release occurred without laser irradiation (Fig. 2i). These results indicated the melt of thermal-responsive liposome shell for on-demand cargo release.

### In vitro evaluation of CDT effect and ICD induction

**Fig. 3:**
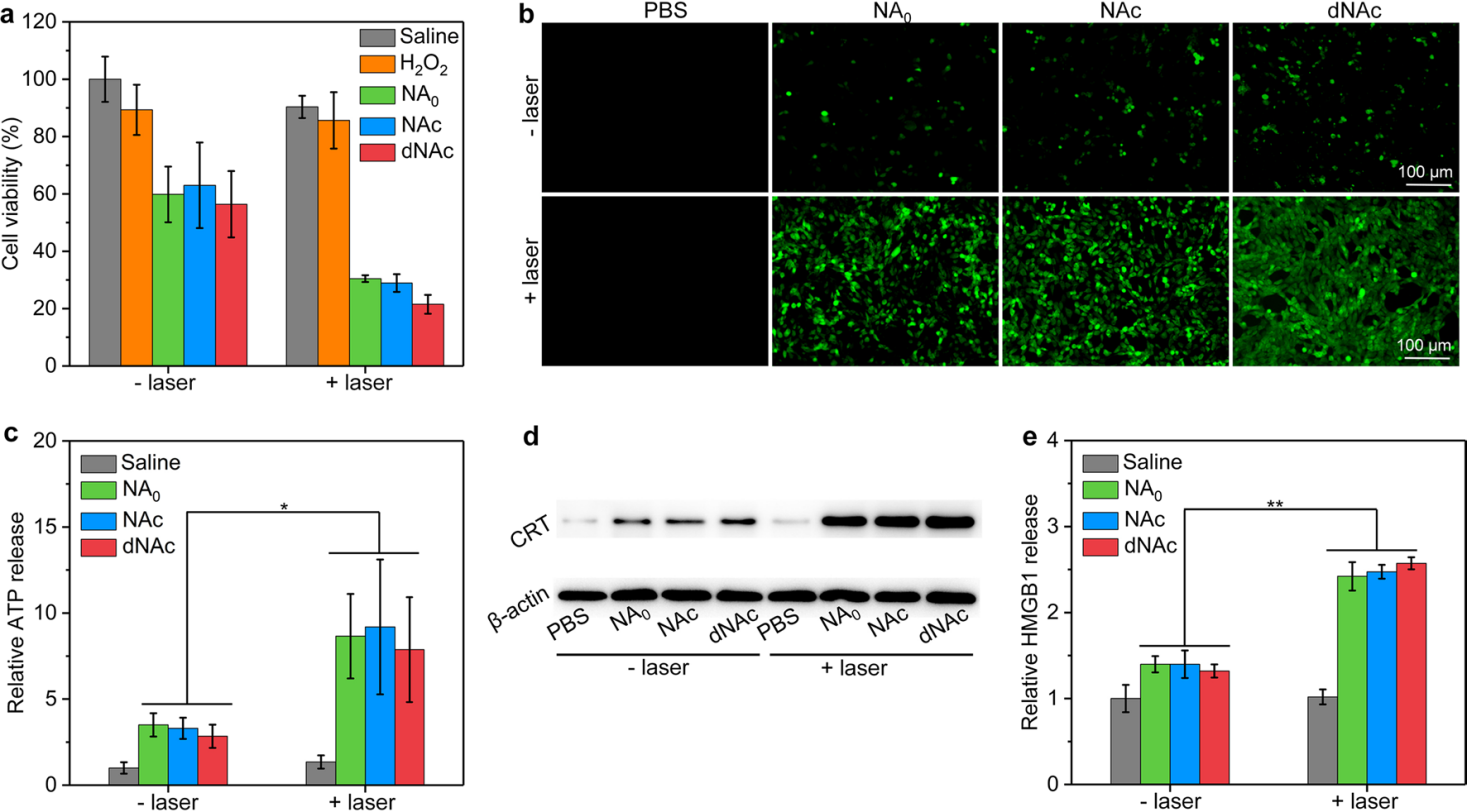
In vitro CDT effect and ICD induction. **a** Cell viability of saline, H_2_O_2_ (100 μM), NA_0_, NAc or dNAc (Fe concentration = 50 μg/mL) treated 4T1 cancer cells in the absence or presence of H_2_O_2_ (100 μM), followed by treatment of NIR-II laser (1 W/cm^2^) for 6 min. **b** Fluorescence images of 4T1 cancer cells treated with NA_0_, NAc or dNAc (50 μg/mL) in the presence of H_2_O_2_ (100 μM), followed by treatment of NIR-II laser (1 W/cm^2^) for 6 min (the generated ·OH showed green fluorescence signals). **c** Extracellular ATP levels for 4T1 cancer cells after different treatments. **d** WB assay of the expression level of CRT for 4T1 cancer cells after different treatments. **e** Detection of HMGB1 extracellular release from 4T1 cancer cells after different treatments

To evaluate nanoagonists-mediated in vitro mild NIR-II PTT augmented CDT, cell viability of 4T1 cancer cells was measured. The cell viability did not obviously change after treatment with NA_0_, NAc and dNAc at the studied Fe concentration ranging 20 ~ 100 µg/mL compared to that of saline treated control cells (Additional file 1: Fig. S6), which suggested the good cytocompatibility of these nanoagonists. To evaluate the mild NIR-II PTT augmented CDT efficacy, 4T1 cancer cells were treated with NA_0_, NAc and dNAc (Fe concentration = 50 µg/mL) in the presence of H_2_O_2_ at the final concentration of 100 μM, followed by treatment with NIR-II laser. Note that the cell viability did not have obvious changes after the cells were treated with H_2_O_2_ without nanoagonists regardless of 1064 nm laser irradiation compared to that of saline-treated control cells (Fig. 3a). After treatment with nanoagonists and H_2_O_2_, the cell viability of cancer cells was significantly reduced due to the generation of highly toxic ·OH for CDT. The cell viabilities for NA_0_, NAc and dNAc-treated cells in the presence of H_2_O_2_ were almost the consistent, suggesting the similar CDT efficacy for these nanoagonists. Furthermore, treatment with NIR-II laser led to lower cell viability compared to that without laser irradiation. A fluorescent indicator for reactive oxygen species (ROS), cell permeant H_2_DCFDA was utilized to evaluate the production of ·OH in cancer cells after treatment. Note that very low fluorescence signals could be observed for 4T1 cancer cells only after treatment with nanoagonists without addition of H_2_O_2_ (Additional file 1: Fig. S7). In contrast, detectable green fluorescence signals were observed for NA_0_, NAc and dNAc-treated cells after the addition of H_2_O_2_ (Fig. 3b). More importantly, NA_0_, NAc and dNAc-treated cells with NIR-II laser irradiation showed much brighter green fluorescence signals than those without laser irradiation, confirming more ·OH generation via mild NIR-II PTT effect. These results verified that mild NIR-II PTT could improve the CDT efficacy to kill cancer cells.

FeS_2_ nanoparticles in the nanoagonists could mediate CDT and mild NIR-II PTT effect under 1064 nm laser irradiation, and such FeS_2_-mediated synergetic action of CDT/NIR-II PTT would induce ICD of dying cancer cells with the release of damage-associated molecular patterns (DAMPs) [71, 72]. To evaluate the generation of ICD biomarkers, 4T1 cancer cells were treated with NA_0_, NAc and dNAc (Fe concentration = 50 µg/mL) in the presence of H_2_O_2_ (100 μM), and then treated by NIR-II laser. The extracellular ATP levels in nanoagonists and H_2_O_2_ treatment groups with laser irradiation were much higher relative to that in saline and sole nanoagonist-treated groups (Fig. 3c). Obviously upregulated expression of CRT was observed for 4T1 cancer cells after treatments with nanoagonists and H_2_O_2_ plus NIR-II laser compared to that of the control cells, while much lower expression of CRT was found for sole nanoagonist-treated cells (Fig. 3d). The extracellular release of HMGB1 from 4T1 cells was found to significantly increase after treatment with nanoagonists in the presence of H_2_O_2_ plus NIR-II laser (Fig. 3e). These results verified the induction of ICD via mild NIR-II PTT augmented CDT using nanoagonists.

### In vivo evaluation of ICD and DC maturation

**Fig. 4:**
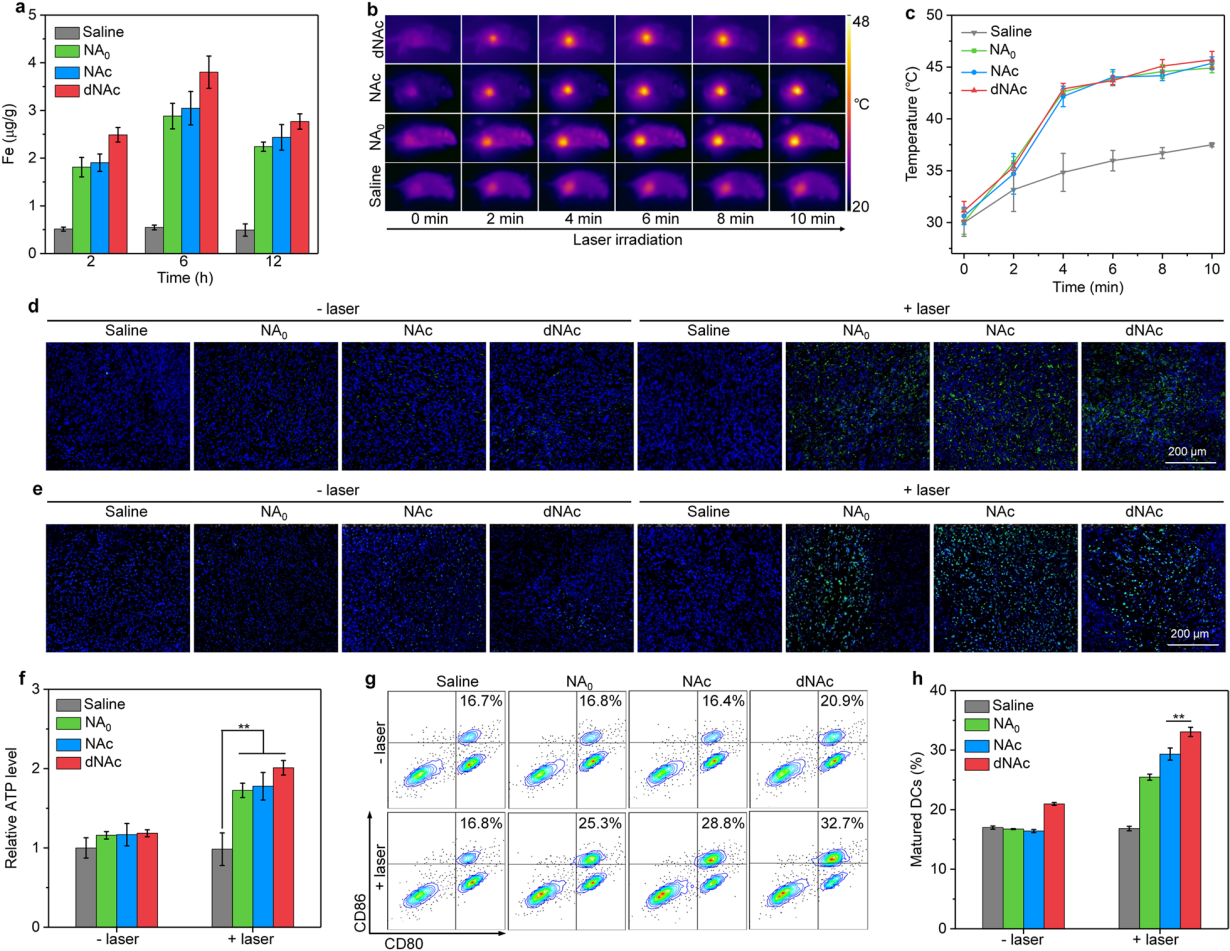
In vivo ICD and DC maturation. **a** Fe content assay in tumors of mice after intravenous injection of saline, NA_0_, NAc and dNAc at different timepoints. **b** Thermal imaging of mice after injection of saline, NA_0_, NAc and dNAc with treatment of NIR-II laser (1 W/cm^2^) for different time. **c** Temperature of tumors for NA_0_, NAc and dNAc-injected mice at different time-points with treatment of NIR-II laser (1 W/cm^2^). Immunofluorescence staining images of CRT (**d**) and HMGB1 (**e**) of tumors. CRT and HMGB1 stained with corresponding antibody showed green fluorescence signals. **f** Relative ATP levels in tumors of mice after intravenous injection of NA_0_, NAc and dNAc with or without treatment of NIR-II laser (1 W/cm^2^) for 10 min. **g** Flow cytometry assay of matured DCs in tumor-draining lymph nodes. **h** Populations of matured DCs (CD80^+^CD86^+^) in tumor-draining lymph nodes

To evaluate the tumor accumulation of nanoagonists, nanoagonists were intravenously injected into 4T1 tumor-bearing mice and Prussian blue staining of tumors was conducted. Nearly no Fe elements were detected in the tumors of mice after saline injection, while obvious Fe elements could be found after the injection of NA_0_, NAc and dNAc at 6 h post-injection timepoint (Additional file 1: Fig. S8), suggesting effective accumulation of nanoagonists in tumor tissues. The Fe contents in tumors at different post-injection timepoints were then measured using ICP-OES. Negligible Fe was detected in tumors of saline-injected mice, while the Fe contents in tumors after injection of nanoagonists were found to increase and reach the maximum at 6 h in a similar manner (Fig. 4a). At 6 h post-injection timepoint, the Fe content in tumors of dNAc-injected group was slightly higher relative to that of NA_0_ and NAc-injected groups, which should be because the surface modified bromelain degraded tumor ECM to facilitate accumulation and diffusion of nanoagonists [73]. The maximum accumulations of all nanoagonists were observed at 6 h post-injection timepoint, possibly because their rapid exclusion from tumor tissues, which was similarly observed for PEG modified FeS_2_ nanoparticles [64]. To verify NIR-II PTT property, after intravenous injection of nanoagonists, tumors of mice were treated by NIR-II laser at the power intensity of 1 W/cm^2^ in a discontinuous manner for 10 min. The tumor temperatures for mice after intravenous injection of NA_0_, NAc and dNAc gradually increased as laser irradiating time, while which for saline injected group did not obviously increase (Fig. 4b). The maximum temperature of tumors was maintained at ~ 45 °C via adjusting the continuity of laser (Fig. 4c).

To confirm the in vivo induction of ICD by mild NIR-II PTT-augmented CDT, the release of ATP and expressions of CRT and HMGB1 in tumors after treatments were investigated. The expression levels of CRT and HMGB1 in tumors were confirmed by immunofluorescence staining. As observed from fluorescence images, obvious expression of CRT could be detected in tumor tissues of mice after injection of nanoagonists with treatment of NIR-II laser, while much lower CRT expression was observed for nanoagonist-injected mice without laser irradiation (Fig. 4d). Similar results were observed for HMGB1, and the expression levels in nanoagonist-injected and laser irradiated groups were significantly higher than those of nanoagonist-injected groups without laser irradiation and control groups (Fig. 4e). After injection of NA_0_, NAc, and dNAc without treatment of NIR-II laser, the ATP levels in tumors only slightly increased compared to the saline-injected control groups (Fig. 4f). In contrast, the relative ATP levels for nanoagonist-injected mice after NIR-II treatment were much higher relative to that of injected mice without laser treatment. These data verified that mild NIR-II PTT-augmented CDT using dNAc could induce ICD with release of DAMPs. Both ICD biomarkers and STING agonists have been reported to promote the maturation of DCs [23, 58]. After different treatments, the maturation of DCs was evaluated using flow cytometry assay. In tumor-draining lymph nodes, the populations of matured DCs (CD80^+^CD86^+^) of mice after injection of nanoagonists and treatment of NIR-II laser were overall higher than that of injected mice without laser treatment and control mice (Fig. 4g). In the dNAc injection and laser irradiation treatment group, the population of matured DCs was the highest, which was 1.14- and 1.96-fold higher relative to that in NAc injected and laser irradiated group and saline-injected control group, respectively (Fig. 4h).

### In vivo antitumor and anti-metastasis evaluation

**Fig. 5:**
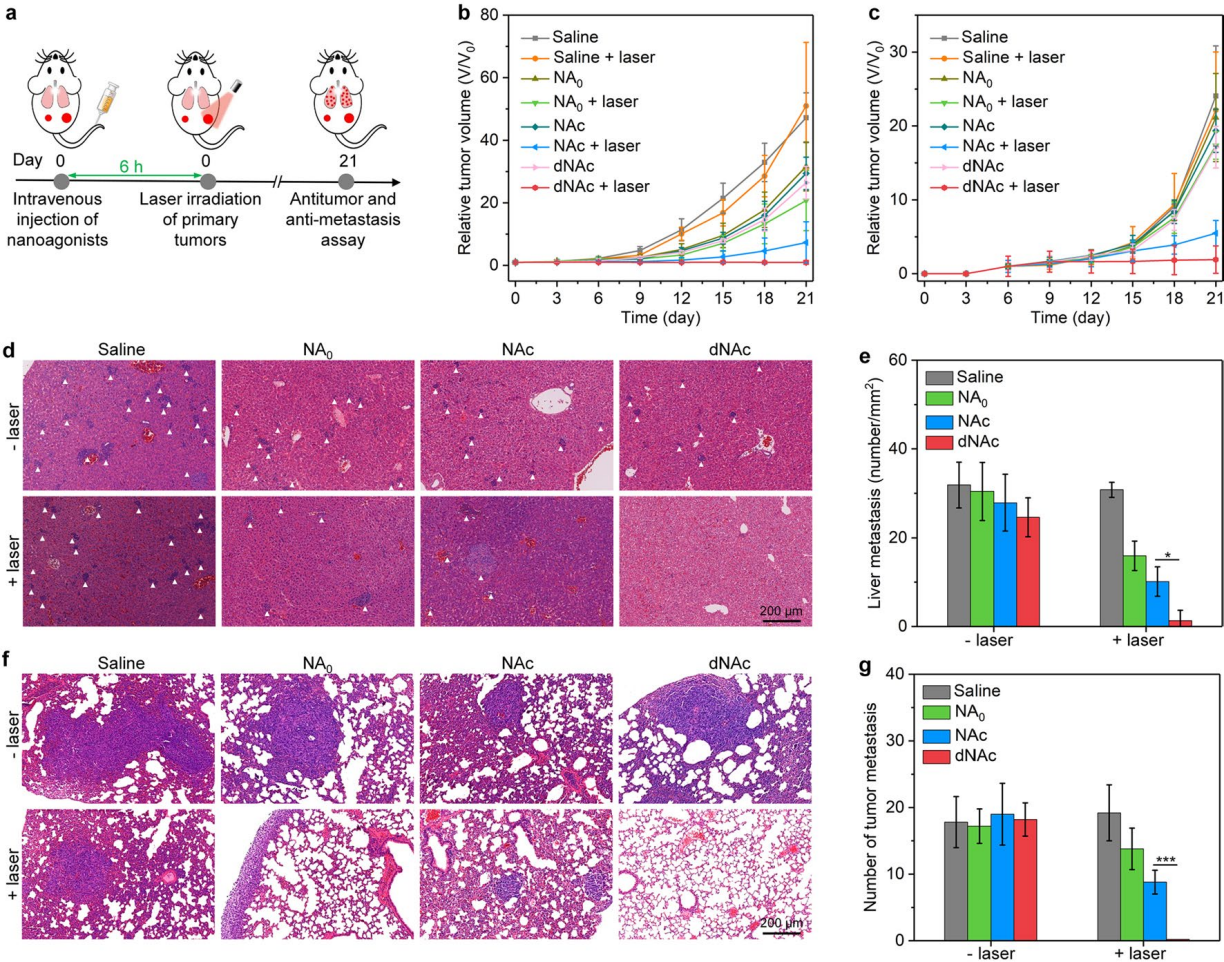
In vivo antitumor and anti-metastasis evaluation. **a** Schematic of intravenous injection of nanoagonists into mice, laser treatment of primary tumors, and evaluation of therapeutic efficacy. Relative tumor volumes of primary tumors (**b**) and distant tumors (**c**) after intravenous injection of NA_0_, NAc and dNAc into mice with or without NIR-II laser treatment (1 W/cm^2^) for 10 min. **d** H&E staining images of livers from mice after nanoagonist injection with or without NIR-II laser treatment (1 W/cm^2^) for 10 min. The white triangles indicated metastatic tumor nodules. **e** Quantification of liver metastasis of mice in different groups. **f** H&E staining images of lungs from mice. **g** Quantification of metastatic tumor nodules in lung of mice

The feasibility of dNAc for mild NIR-II PTT augmented CDT-immunotherapy was evaluated using bilateral 4T1 tumor-bearing BALB/c mouse models with NA_0_ and NAc as control counterparts. The primary tumors of mice were treated by NIR-II laser (1 W/cm^2^) at 6 h post-injection of nanoagonists (Fig. 5a). After treatments, the growth of primary tumors of NA_0_, NAc and dNAc-injected mice without laser irradiation was only slightly inhibited compared to control mice (Fig. 5b), while the tumor growth was significantly inhibited for nanoagonist-injected mice after treatment of laser. Note that the inhibitory efficacy of primary tumors in dNAc-injected group with treatment of laser was 97.9%, which was much higher relative to that in NA_0_ (79.6%) and NAc (84.5%) injected group with laser irradiation. Nanoagonist-mediated therapy after NIR-II laser treatment also greatly inhibited the growth of distant tumors, while sole nanoagonist treatment without laser irradiation did not show remarkable inhibition efficacy (Fig. 5c). The inhibitory efficiency of tumors in dNAc-treated and laser treatment group was 92.1%, which was 3.3- and 1.2-fold higher relative to that in NA_0_ and NAc-treated and laser irradiated group, respectively. H&E and TUNEL staining showed that the severest cell necrosis and apoptosis in primary and distant tumors was observed for dNAc-injected laser irradiated group (Fig. S9-10, Supporting information). In addition, the expression of Ki67 in dNAc-treated tumors with NIR-II laser treatment was much lower relative to that in the other groups (Additional file 1: Fig. S11). These results suggested that dNAc-mediated therapy could suppress primary and distant tumor growths. Note that the therapeutic efficacy of cGAMP was limited [38], which should be due to its poor accumulation in tumor sites and lacking of direct cancer cell killing capability. Although delivery of STING agonists into tumor sites could be achieved using nanoplatforms, the multiple injection of agonist-loaded nanoparticles was often needed for effective cancer immunotherapy [38, 74], suggesting the limited therapeutic efficacy for sole immunotherapy. In our present study, only a single injection could result in ideal antitumor effect, which verified the use of STING agonists in a safer and more effective manner. The tumor inhibitory efficacy of dNAc was higher than that of previously reported PEG modified FeS_2_ nanoparticles that only exerted NIR-II PTT and CDT [64], which should be due to the combinational action of NIR-II PTT, CDT and immunotherapy. To obtain a similar antitumor effect, a much higher photothermal temperature (51 °C) was required for semiconducting polymer nanoadjuvant [58] when compared to the mild temperature (~ 45 °C) in this study.

The anti-metastasis efficacy of nanoagonist-mediated therapy was then evaluated using H&E staining. Tumor metastases could be observed in livers of mice from all groups, except for dNAc injected and laser irradiated group (Fig. 5d). Nanoagonist-mediated therapy greatly reduced liver metastases in different degrees, and the least liver metastases were observed for dNAc injected and laser irradiated group (Fig. 5e). In addition, nearly no metastatic lesions were observed in lungs of dNAc-treated and laser irradiated mice, in contrast, tumor metastases were detected in lung tissues of mice after the other treatments (Fig. 5f). The metastasis nodules in lung of dNAc-treated and laser treated group was much less relative to those in the other treated groups (Fig. 5g). Therefore, dNAc had the highest therapeutic efficacy in preventing both liver and lung metastasis.

### In vivo evaluation of antitumor immune response

**Fig. 6:**
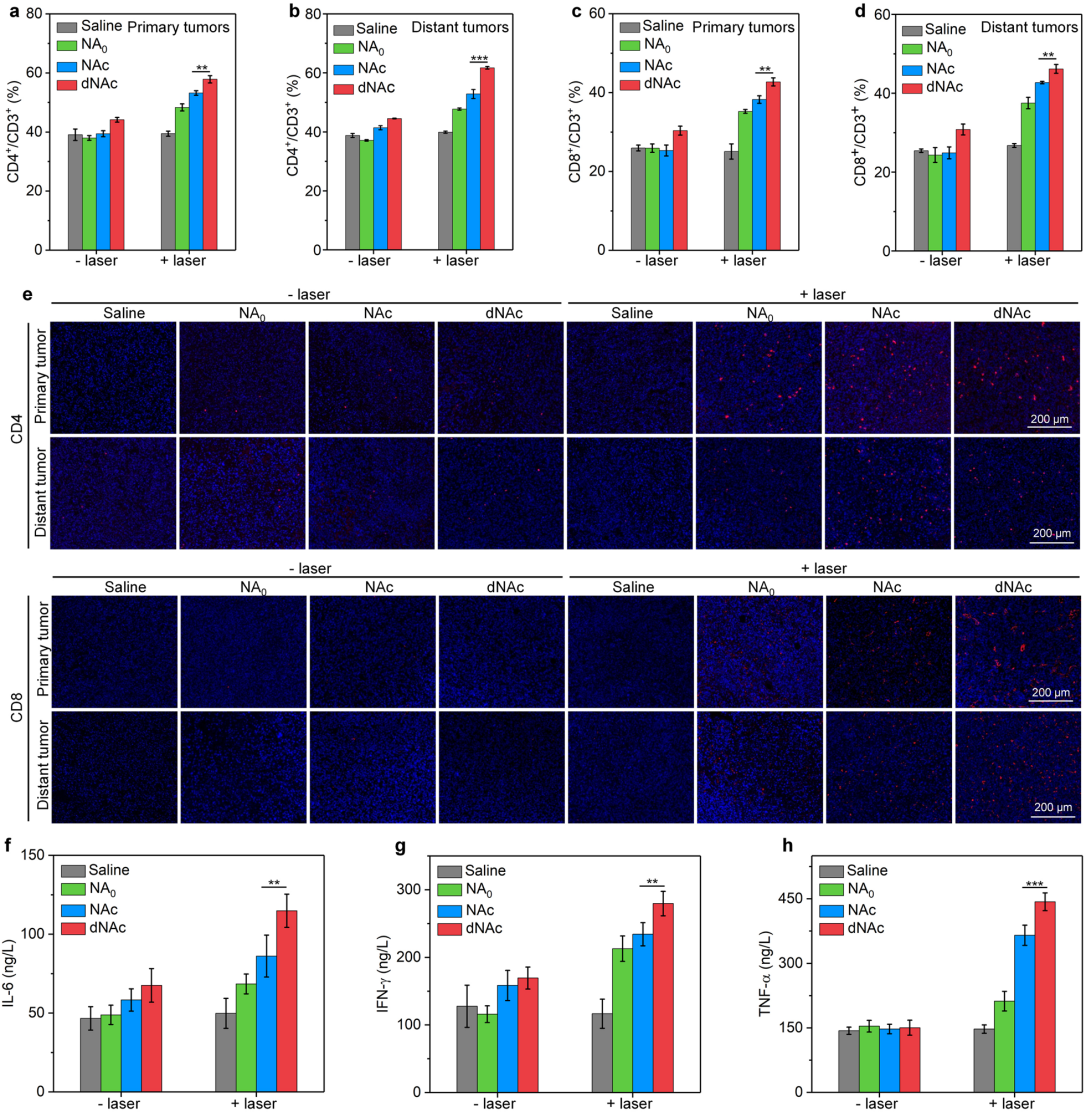
Mechanism of in vivo antitumor efficacy. The percentages of CD3^+^CD4^+^ T cells in primary tumors (**a**) and distant tumors (**b**) after intravenous injection of nanoagonists into mice with or without treatment of NIR-II laser (1 W/cm^2^) for 10 min. The percentages of CD3^+^CD8^+^ T cells in primary tumors (**c**) and distant tumors (**d**) from different treated mice. **e** Immunofluorescence staining images of CD4 and CD8 (red fluorescence signals) in primary and distant tumors. Serum levels of IL-6 (**f**), IFN-γ (**g**) and TNF-α (**h**) after different treatments of mice

The percentages of CD3^+^CD4^+^ and CD3^+^CD8^+^ T cells in tumors were then evaluated to verify the activation of antitumor immunity. In both primary and distant tumors, the percentages of CD3^+^CD4^+^ T cells were significantly increased after treatment with nanoagonists and NIR-II laser irradiation, while which in nanoagonist-treated groups without laser irradiation did not have obvious increment (Fig. 6a, b and Additional file 1: Fig. S12). In dNAc-injected and laser irradiated group, the population of CD3^+^CD4^+^ T cells was at least 1.1- and 1.2-fold higher relative to that in the other groups. The nanoagonist treatment and laser irradiation also increased the percentages of CD3^+^CD8^+^ T cells in tumors of living mice, and the highest populations of CD3^+^CD8^+^ T cells were observed for mice after treatment with dNAc and laser irradiation (Fig. 6c, d and Additional file 1: Fig. S13). In dNAc plus laser irradiation group, the percentages of CD3^+^CD8^+^ T cells in primary and distant tumors was 1.6- and 1.8-fold higher relative to that for control group, respectively. These results suggested that dNAc-mediated mild photothermal effect could trigger on-demand release of cGAMP to promote priming of effector T cells. Higher percentages of CD3^+^CD4^+^ and CD3^+^CD8^+^ T cells were found in tumors, which should also be due to the fact that dNAc-enabled degradation of ECM could facilitate the infiltration of T cells into tumor tissues [75].

Immunofluorescence staining was then conducted to evaluate intratumor T cells. More CD4^+^ and CD8^+^ T cells could be observed in both primary and distant tumors after treatment of nanoagonists plus NIR-II laser relative to those after treatments with nanoagonists without laser irradiation and those in control groups (Fig. 6e). The dNAc-injected and laser irradiated group showed the highest fluorescence signals for CD4 and CD8 staining among the treated groups. The contents of immune-relevant cytokines were measured to confirm the immune activation. The highest serum level of IL-6, IFN-γ and TNF-α was observed in dNAC injection plus laser irradiation group, which was at least 1.3-, 1.2-, and 1.2-fold higher relative to that in the other treated groups, respectively (Fig. 6f–h).

### In vivo evaluation of biosafety

As one of the major components of nanoagonists, the toxicity, biodistribution and excretion of BSA-FeS_2_ nanoparticles were first evaluated. After intravenous injection of BSA-FeS_2_ nanoparticles into 4T1 tumor-bearing mice, no histological damages were observed for heart, liver, spleen, lung and kidney (Additional file 1: Fig. S14). The serum levels of alkaline phosphatase (ALP), alanine aminotransferase (ALT), aspartate aminotransferase (AST), blood urea nitrogen (BUN), and creatinine (CREA) in mice did not have obvious changes after injection of BSA-FeS_2_ nanoparticles (Additional file 1: Fig. S15). These results suggested the negligible toxicity of BSA-FeS_2_ nanoparticles. The biodistribution was evaluated using ICP-OES, and BSA-FeS_2_ nanoparticles were found to mainly accumulate into liver and spleen, which are important metabolic organs to rapidly clear foreign matters by phagocyte (Additional file 1: Fig. S16). In addition, the accumulation of BSA-FeS_2_ nanoparticles in all tissues was significantly reduced after 7 days of injection, which indicated the excretion of BSA-FeS_2_ nanoparticles from living bodies.

To evaluate in vivo biosafety of nanoagonists for cancer therapy, 4T1 tumor-bearing mice were injected with nanoagonists, and the primary tumors were treated by NIR-II laser for 10 min. After treatments for 21 days, the body weights of nanoagonist-injected mice regardless of laser irradiation did not have obvious changes (Additional file 1: Fig. S17). H&E staining images showed that the histological morphologies of heart, spleen and kidney in the treated mice were almost the same to those of control mice (Additional file 1: Fig. S18). The results of blood biochemistry assay showed that the liver/kidney function indicators and various vital blood parameters in serum did not have negligible differences between treated and control mice (Additional file 1: Fig. S19). Thus, dNAc at the studied injection dosage (Fe concentration = 300 μg/mL) not only led to an ideal antitumor efficacy, but also showed good in vivo biosafety because no damages of normal tissues were observed. However, the long-term biosafety of dNAc in large animals at different injection dosages should be systematically investigated by evaluating the balance of Fe metabolism under normal physiological conditions to ensure effective and safe concentration for the further clinical practical applications.

## Conclusion

We have developed an ECM-degrading nanoagonist that can achieve remote on-demand release of agonists upon NIR-II photoirradiation to activate STING pathway for mild PTT-augmented CDT-immunotherapy. As a major component within dNAc, FeS_2_ nanoparticles mediated Fenton reaction to generate ·OH, which would be enhanced by mild photothermal effect under NIR-II laser treatment, leading to death of tumor cells and induction of ICD. Precise release of STING agonists in tumors was achieved due to the destruction of thermal-responsive liposome shell caused by generated heat, allowing for photoactivation of STING pathway. The combinational action of ICD and activation of STING pathway facilitated DC maturation and effector T cell priming. Thanks to ECM degradation in tumor microenvironment by enzyme on dNAc surface, the infiltration of T cells into tumors was greatly improved, leading to amplified antitumor immune response. As such, both primary and distant tumors were significantly cured and tumor metastasis in livers and lungs were nearly completely restrained after dNAc-mediated therapy in a mouse tumor model.

On-demand release of STING agonists is the most important for cancer immunotherapy in a safe and effective manner, and we for the first time report the use of photothermal nanoplatforms to achieve this purpose. The mild NIR-II photothermal-augmented CDT effect that can ablate tumors, induce ICD, and modulate the immunosuppressive tumor microenvironment, and the degradation of tumor ECM to promote T cell infiltration are also pivotal for amplified cancer immunotherapy. This study thus demonstrates an ECM-degrading nanoagonist with mild NIR-II photothermal activated STING pathway and improved Fenton reaction efficacy for enhanced cancer CDT-immunotherapy.

## Supplementary Information

**Additional file 1: Fig. S1.** TGA analysis of FeS_2_, BSA-FeS_2_, NA_0_, NAc and dNAc. **Fig. S2.** Photographs of aqueous solutions of NA_0_, NAc and dNAc after storage for 2 weeks. **Fig. S3.** (a) Photographs of red blood cells after treatments with 1% Triton X-100 (+ control), PBS buffer (− control), and NA_0_, NAc or dNAc at different concentrations (7.5, 15, 30, 60, and 120 μg/mL) for 2 h. (b) Hemolysis assay of red blood cells after treatments with NA_0_, NAc or dNAc at different concentrations for 2 h. **Fig. S4.** Thermal imaging of NA_0_, NAc and dNAc as a foundation of laser irradiation time under NIR-II laser irradiation at the power density of 1 W/cm^2^. **Fig. S5.** (a) Thermal imaging of dNAc solutions covered with chicken breast tissue of different thicknesses (0, 1, 3, 5, and, 7 mm) as a foundation of laser irradiation time under NIR-II laser irradiation at the power density of 1 W/cm^2^. (b) Temperature curves of dNAc covered with chicken breast tissue of different thicknesses (0, 1, 3, 5, and 7 mm) after treatment of NIR-II laser (1 W/cm^2^) for different time. (c) Temperature increment (ΔT) of dNAc covered with chicken breast tissue of different thicknesses (0, 1, 3, 5, and 7 mm) after treatment of NIR-II laser (1 W/cm^2^) for different time. **Fig. S6.** Cell viability of 4T1 cancer cells after incubation with NA_0_, NAc or dNAc at different concentrations (0, 20, 40, 60, 80, and 100 μg/mL) for 24 h. **Fig. S7.** Confocal fluorescence images of 4T1 cancer cells after treatment with PBS, NA_0_, NAc and dNAc (50 μg/mL) in the absence of H_2_O_2_. **Fig. S8.** Prussian blue staining images of tumor sections from 4T1 tumor-bearing mice after intravenous injection of saline, NA_0_, NAc and dNAc at 6 h post-injection timepoint. Black arrow indicated Prussian blue staining of Fe. **Fig. S9.** H&E staining images of primary tumors (a) and distant tumors (b) from 4T1 tumor-bearing mice after intravenous injection of NA_0_, NAc and dNAc with or without NIR-II laser irradiation. **Fig. S10.** TUNEL staining images of primary tumors (a) and distant tumors (b) from 4T1 tumor-bearing mice after intravenous injection of NA_0_, NAc and dNAc with or without NIR-II laser irradiation. **Fig. S11.** Ki67 staining images of primary tumors (a) and distant tumors (b) from 4T1 tumor-bearing mice after intravenous injection of NA_0_, NAc and dNAc with or without NIR-II laser irradiation. **Fig. S12. **Flow cytometry assay of CD3^+^CD4^+^ T cells in primary tumors (a) and distant tumors (b) of mice after intravenous injection of nanoagonists with or without NIR-II laser irradiation. **Fig. S13. **Flow cytometry assay of CD3^+^CD8^+^ T cells in primary tumors (a) and distant tumors (b) of mice after intravenous injection of nanoagonists with or without NIR-II laser irradiation. **Fig. S14.** H&E staining images of heart, liver, spleen, lung, and kidney of mice after intravenous injection of BSA-FeS_2_ nanoparticles. **Fig. S15.** The serum levels of (a) alkaline phosphatase (ALP), (b) alanine aminotransferase (ALT), (c) aspartate aminotransferase (AST), (d) blood urea nitrogen (BUN) and (e) creatinine (CREA) in mice after intravenous injection of BSA-FeS_2_ nanoparticles. **Fig. S16.** Biodistribution of BSA-FeS_2_ nanoparticles in heart, liver, spleen, lung, kidney, and tumors at different post-injection timepoints. **Fig. S17.** Body weight of 4T1 tumor-bearing mice after intravenous injection of NA_0_, NAc or dNAc with or without NIR-II laser irradiation for different days. **Fig. S18.** H&E staining images of heart, spleen, and kidney of mice after different treatments for 21 days. **Fig. S19.** The level of (a) red blood cells, (b) red cell distribution width, (c) white blood cells, (d) mean corpuscular hemoglobin, (e) mean corpuscular volume, (f) hematocrit, (g) hemoglobin, (h) ALP, (i) ALT, (j) AST, (k) BUN and (l) CREA in blood samples of control mice and mice after dNAc-mediated therapy.

## Data Availability

The datasets used and analyzed during the current study are available from the corresponding author on reasonable request.
