# Supplementary material for: Extracellular matrix-degrading STING nanoagonists for mild NIR-II photothermal-augmented chemodynamic-immunotherapy
Source: J Nanobiotechnology. 2022 Jan 6;20:23. doi: 10.1186/s12951-021-01226-3 (PMC8740364; doi:10.1186/s12951-021-01226-3)
Supplement: Supplementary file 1 — Additional file 1: Fig. S1. TGA analysis of FeS2, BSA-FeS2, NA0, NAc and dNAc. Fig. S2. Photographs of aqueous solutions of NA0, NAc and dNAc after storage for 2 weeks. Fig. S3. (a) Photographs of red blood cells after treatments with 1% Triton X-100 (+ control), PBS buffer (− control), and NA0, NAc or dNAc at different concentrations (7.5, 15, 30, 60, and 120 μg/mL) for 2 h. (b) Hemolysis assay of red blood cells after treatments with NA0, NAc or dNAc at different concentrations for 2 h. Fig. S4. Thermal imaging of NA0, NAc and dNAc as a foundation of laser irradiation time under NIR-II laser irradiation at the power density of 1 W/cm2. Fig. S5. (a) Thermal imaging of dNAc solutions covered with chicken breast tissue of different thicknesses (0, 1, 3, 5, and, 7 mm) as a foundation of laser irradiation time under NIR-II laser irradiation at the power density of 1 W/cm2. (b) Temperature curves of dNAc covered with chicken breast tissue of different thicknesses (0, 1, 3, 5, and 7 mm) after treatment of NIR-II laser (1 W/cm2) for different time. (c) Temperature increment (ΔT) of dNAc covered with chicken breast tissue of different thicknesses (0, 1, 3, 5, and 7 mm) after treatment of NIR-II laser (1 W/cm2) for different time. Fig. S6. Cell viability of 4T1 cancer cells after incubation with NA0, NAc or dNAc at different concentrations (0, 20, 40, 60, 80, and 100 μg/mL) for 24 h. Fig. S7. Confocal fluorescence images of 4T1 cancer cells after treatment with PBS, NA0, NAc and dNAc (50 μg/mL) in the absence of H2O2. Fig. S8. Prussian blue staining images of tumor sections from 4T1 tumor-bearing mice after intravenous injection of saline, NA0, NAc and dNAc at 6 h post-injection timepoint. Black arrow indicated Prussian blue staining of Fe. Fig. S9. H&E staining images of primary tumors (a) and distant tumors (b) from 4T1 tumor-bearing mice after intravenous injection of NA0, NAc and dNAc with or without NIR-II laser irradiation. Fig. S10. TUNEL staining images of primary t [file 12951_2021_1226_MOESM1_ESM.docx]

**Additional Information**

**Extracellular matrix-degrading STING nanoagonists for mild NIR-II photothermal-augmented chemodynamic-immunotherapy**

Meixiao Zhan^1#^, Xiangrong Yu^1#^, Wei Zhao^1^, Yongjun Peng^1^, Shaojun Peng^1^*, Jingchao Li^2^*, Ligong Lu^1^*

^1^ Zhuhai Institute of Translational Medicine, Zhuhai Precision Medical Center, Zhuhai People’s Hospital (Zhuhai hospital affiliated with Jinan University), Zhuhai, Guangdong 519000, China. Email: henry2008_ok@126.com; luligong1969@jnu.edu.cn

^2^ Shanghai Engineering Research Center of Nano-Biomaterials and Regenerative Medicine, College of Chemistry, Chemical Engineering and Biotechnology, Donghua University, Shanghai 201620, China. Email: jcli@dhu.edu.cn

^#^ Meixiao Zhan and Xiangrong Yu contributed equally to this work.


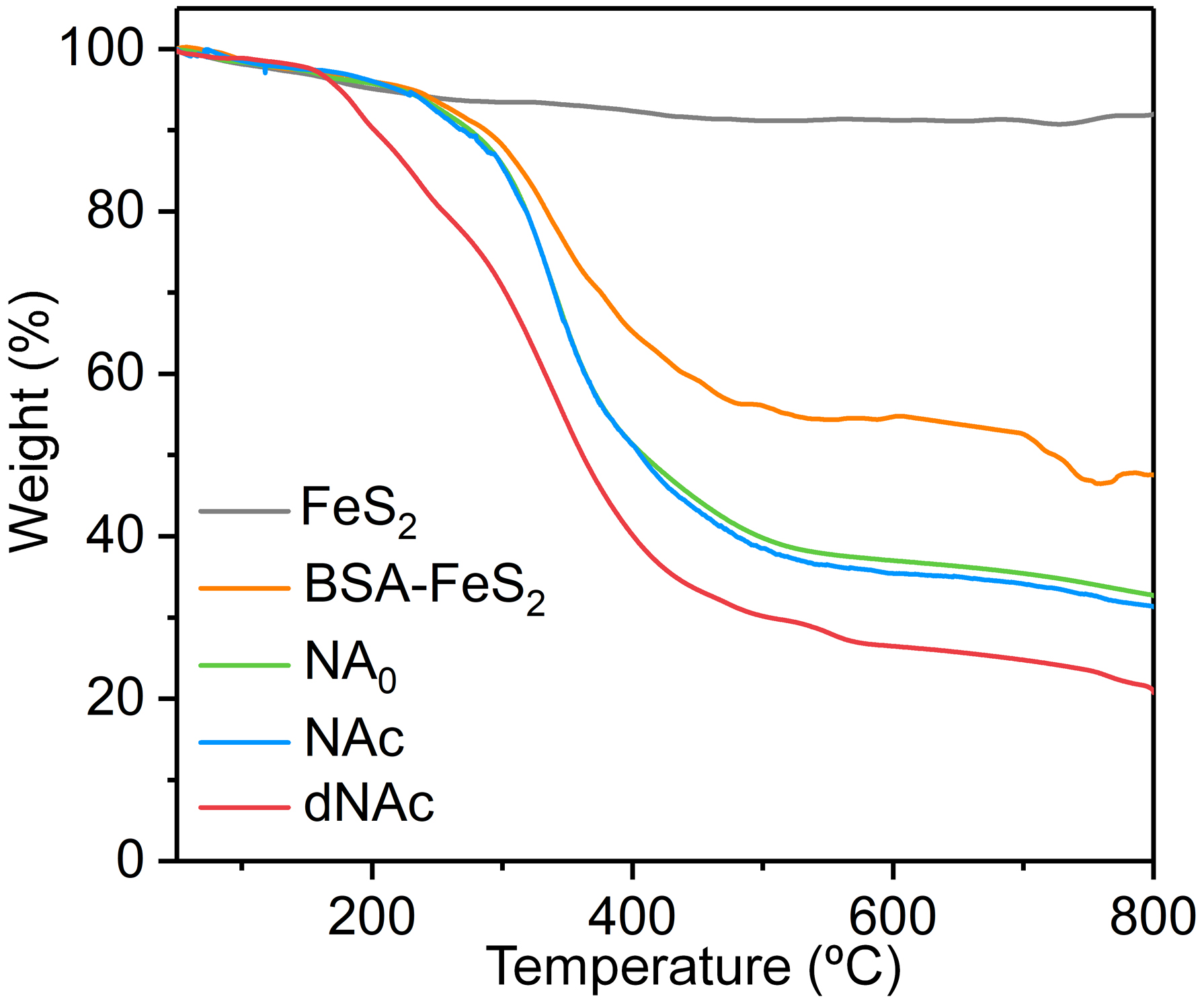


**Fig. S1.** TGA analysis of FeS_2_, BSA-FeS_2_, NA_0_, NAc and dNAc.


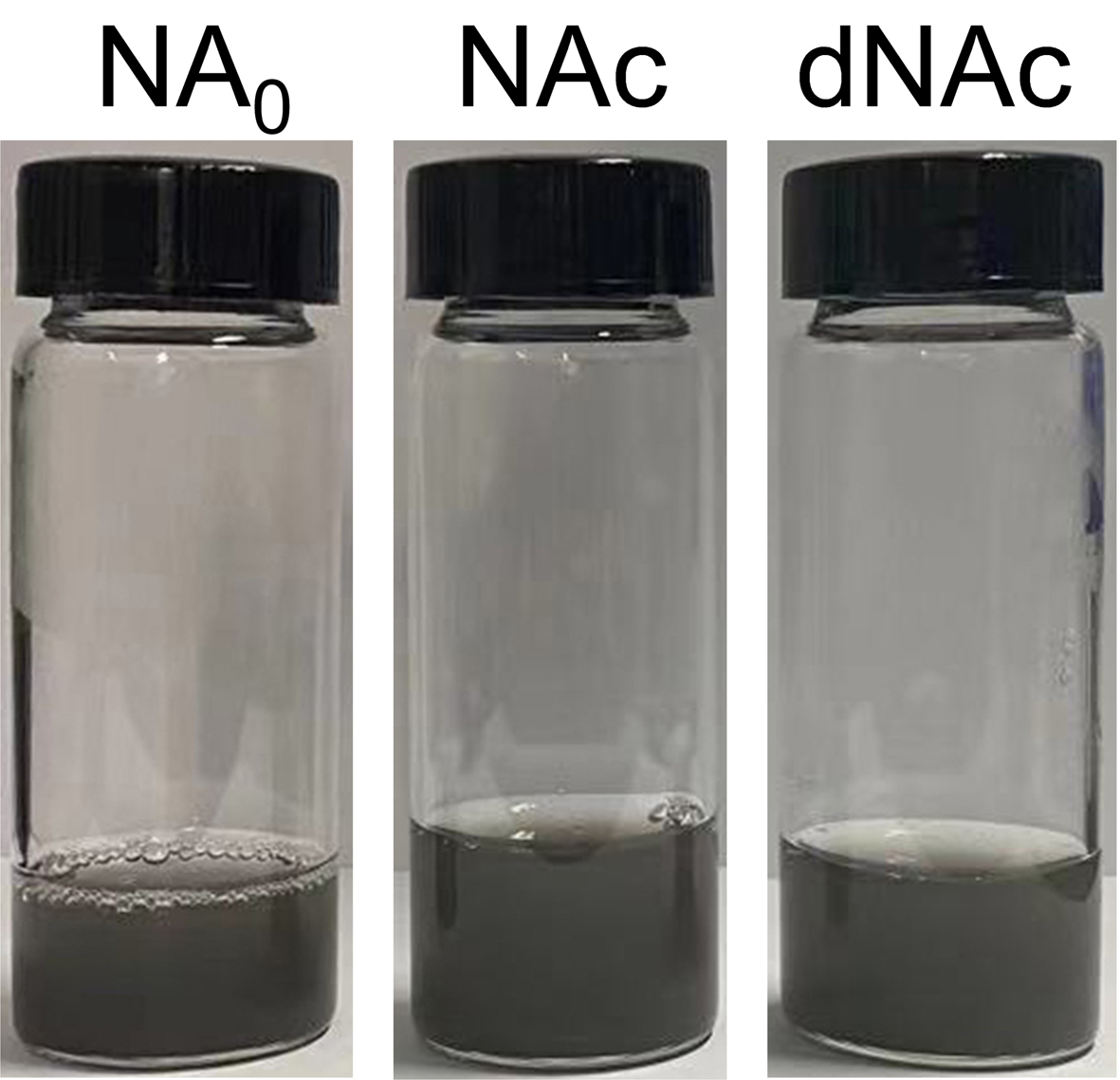


**Fig. S2.** Photographs of aqueous solutions of NA_0_, NAc and dNAc after storage for 2 weeks.


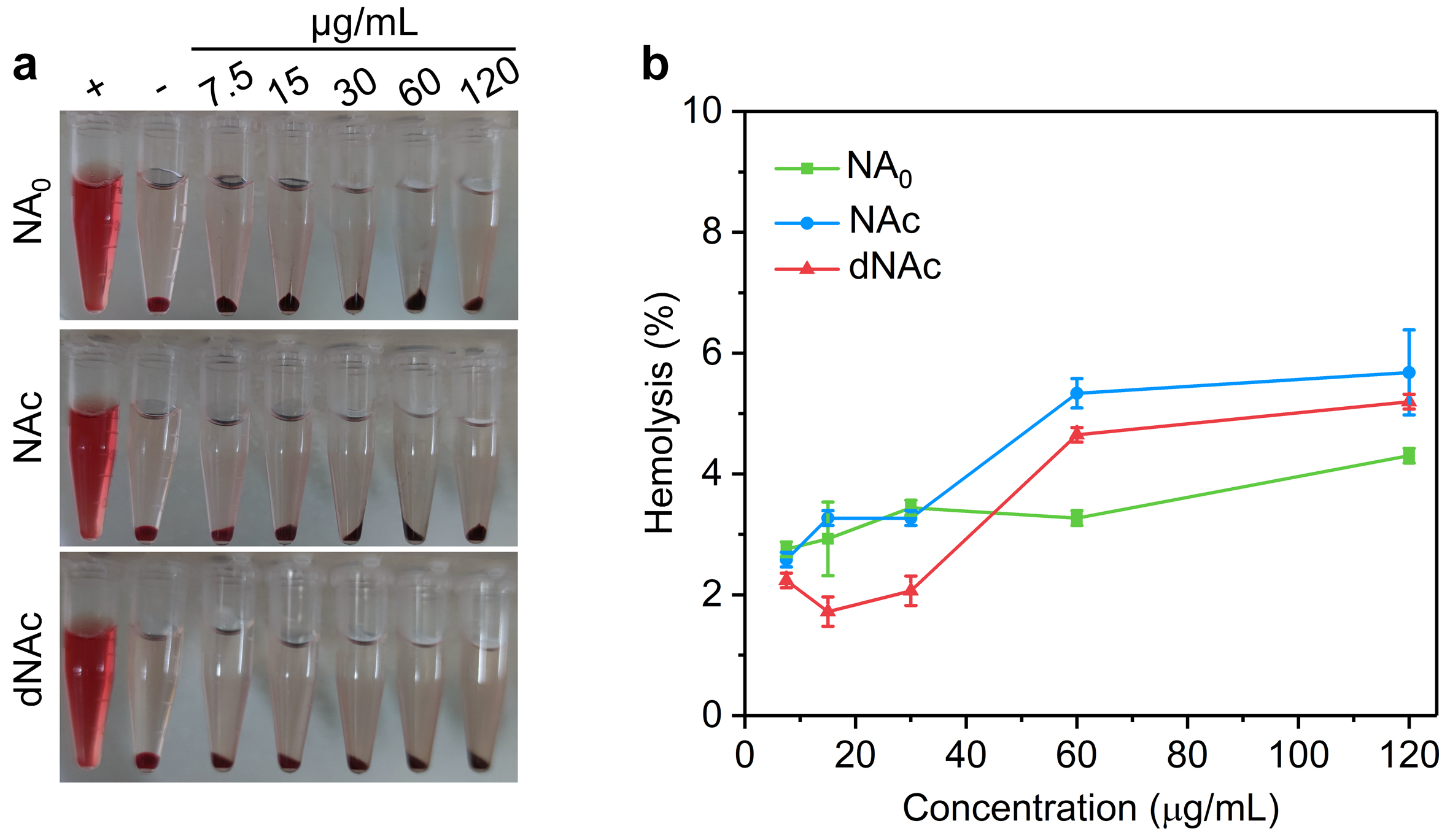


**Fig. S3.** (a) Photographs of red blood cells after treatments with 1% Triton X-100 (+ control), PBS buffer (- control), and NA_0_, NAc or dNAc at different concentrations (7.5, 15, 30, 60, and 120 μg/mL) for 2 h. (b) Hemolysis assay of red blood cells after treatments with NA_0_, NAc or dNAc at different concentrations for 2 h.


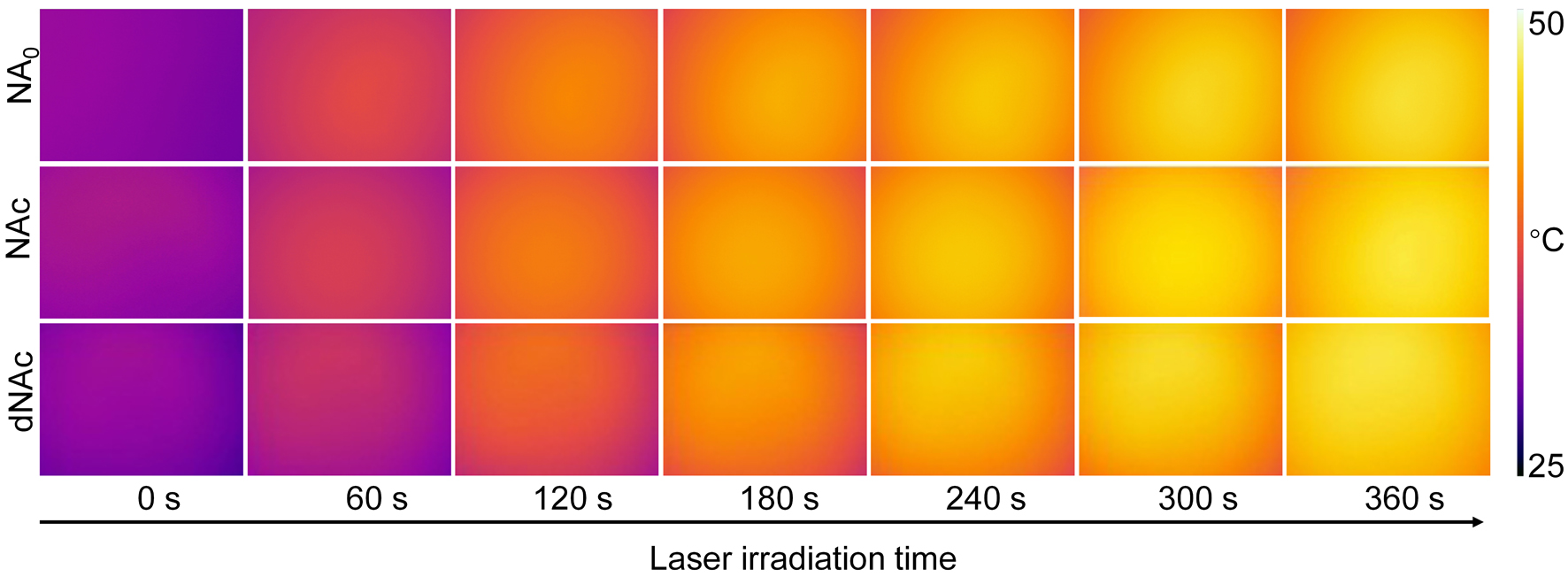


**Fig. S4.** Thermal imaging of NA_0_, NAc and dNAc as a foundation of laser irradiation time under NIR-II laser irradiation at the power density of 1 W/cm^2^.


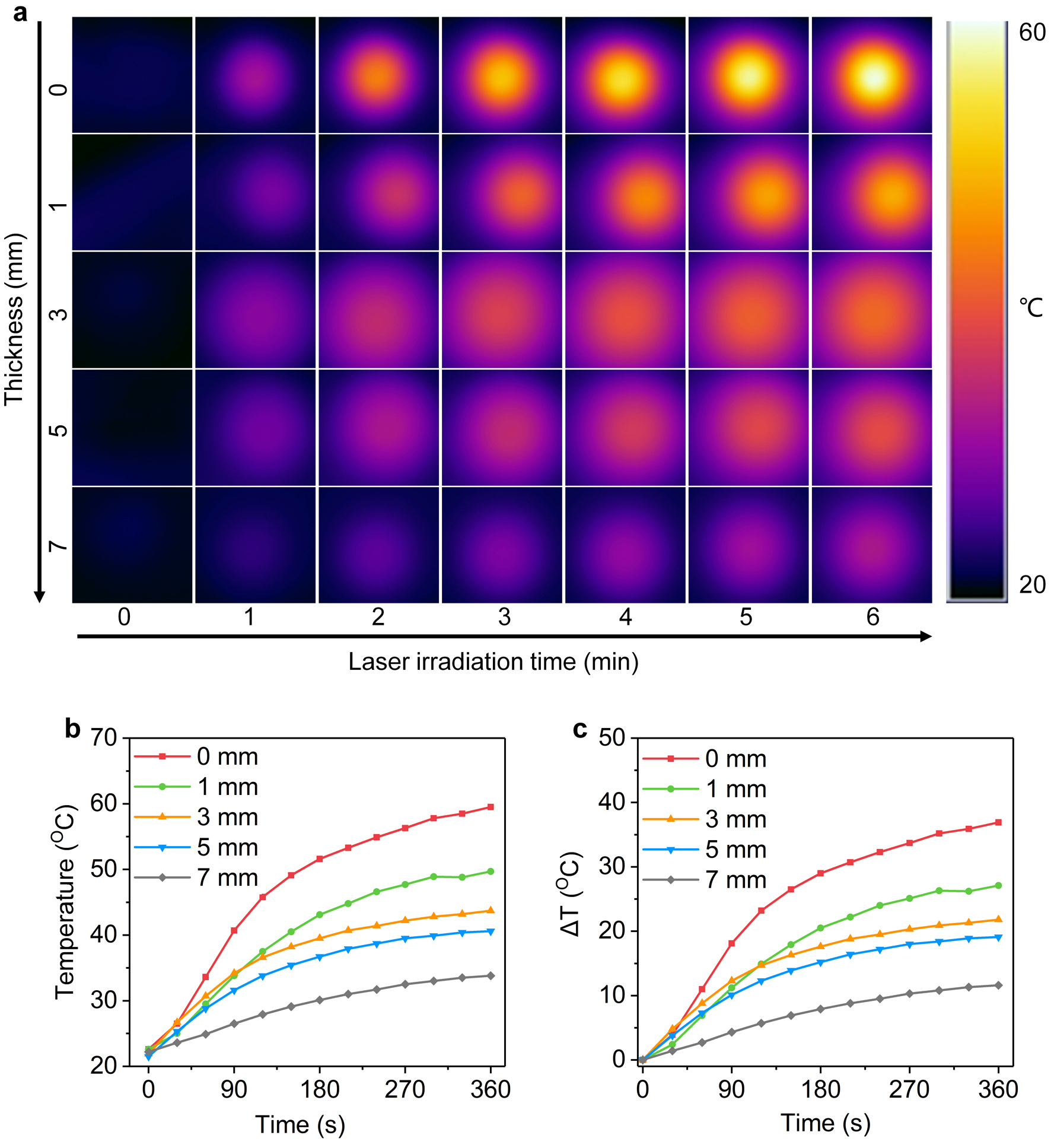


**Fig. S5.** (a) Thermal imaging of dNAc solutions covered with chicken breast tissue of different thicknesses (0, 1, 3, 5, and, 7 mm) as a foundation of laser irradiation time under NIR-II laser irradiation at the power density of 1 W/cm^2^. (b) Temperature curves of dNAc covered with chicken breast tissue of different thicknesses (0, 1, 3, 5, and 7 mm) after treatment of NIR-II laser (1 W/cm^2^) for different time. (c) Temperature increment (ΔT) of dNAc covered with chicken breast tissue of different thicknesses (0, 1, 3, 5, and 7 mm) after treatment of NIR-II laser (1 W/cm^2^) for different time.


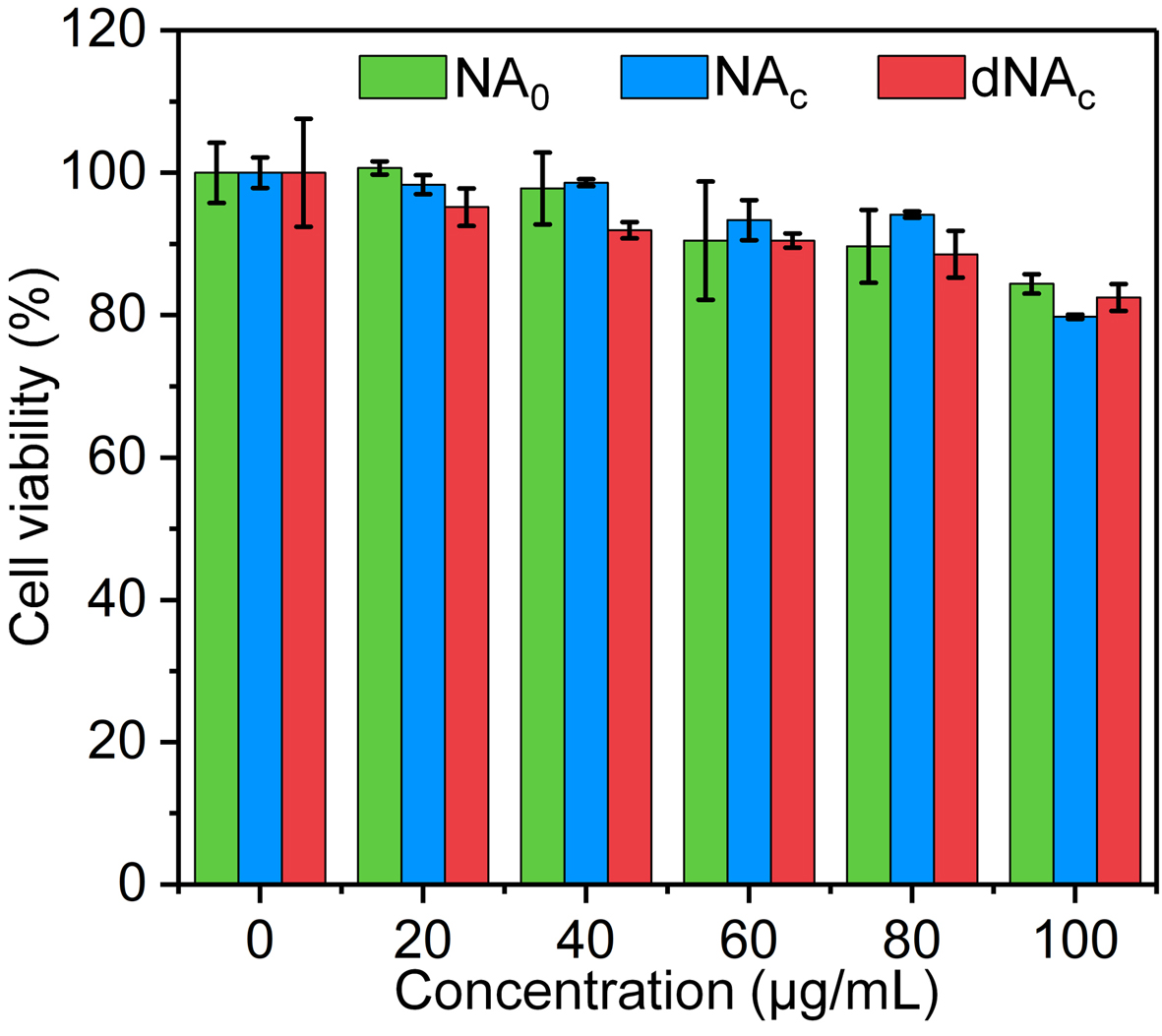


**Fig. S6.** Cell viability of 4T1 cancer cells after incubation with NA_0_, NAc or dNAc at different concentrations (0, 20, 40, 60, 80, and 100 μg/mL) for 24 h.


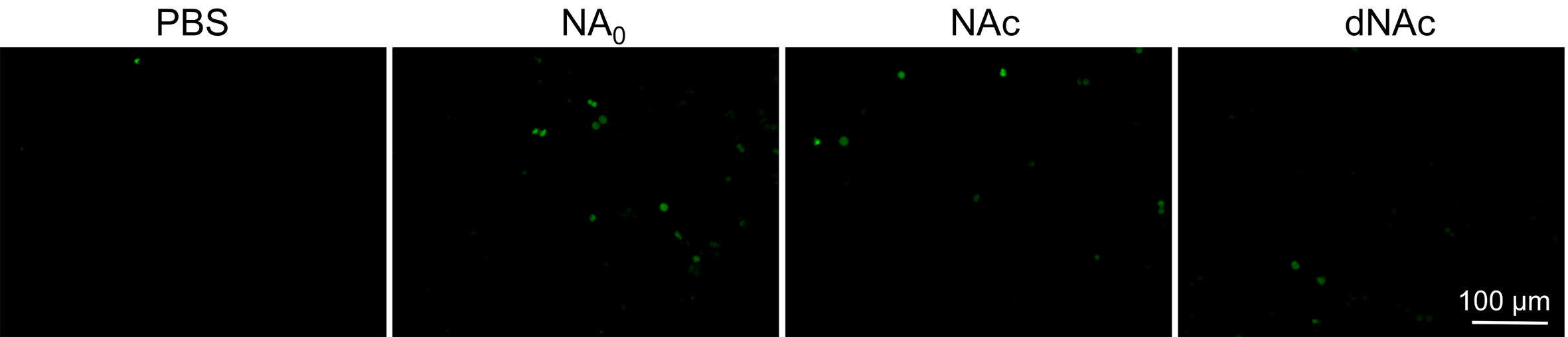


**Fig. S7.** Confocal fluorescence images of 4T1 cancer cells after treatment with PBS, NA_0_, NAc and dNAc (50 μg/mL) in the absence of H_2_O_2_.


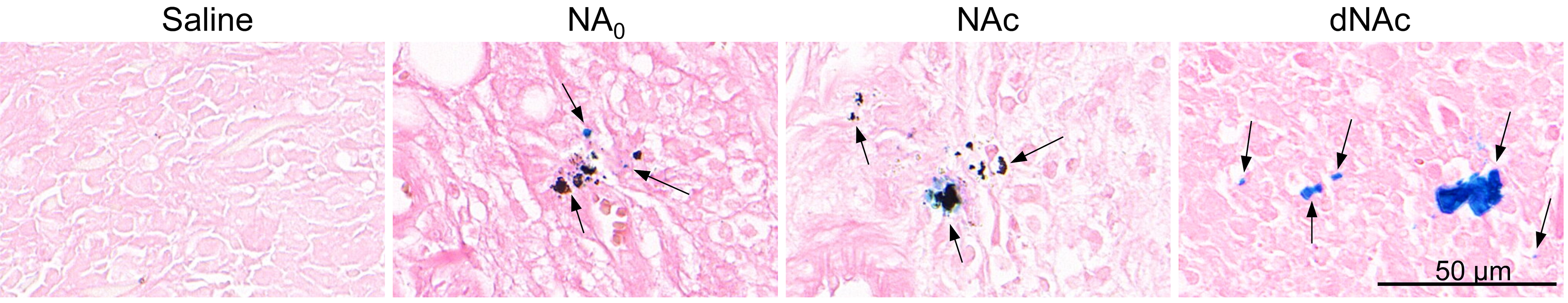


**Fig. S8.** Prussian blue staining images of tumor sections from 4T1 tumor-bearing mice after intravenous injection of saline, NA_0_, NAc and dNAc at 6 h post-injection timepoint. Black arrow indicated Prussian blue staining of Fe.


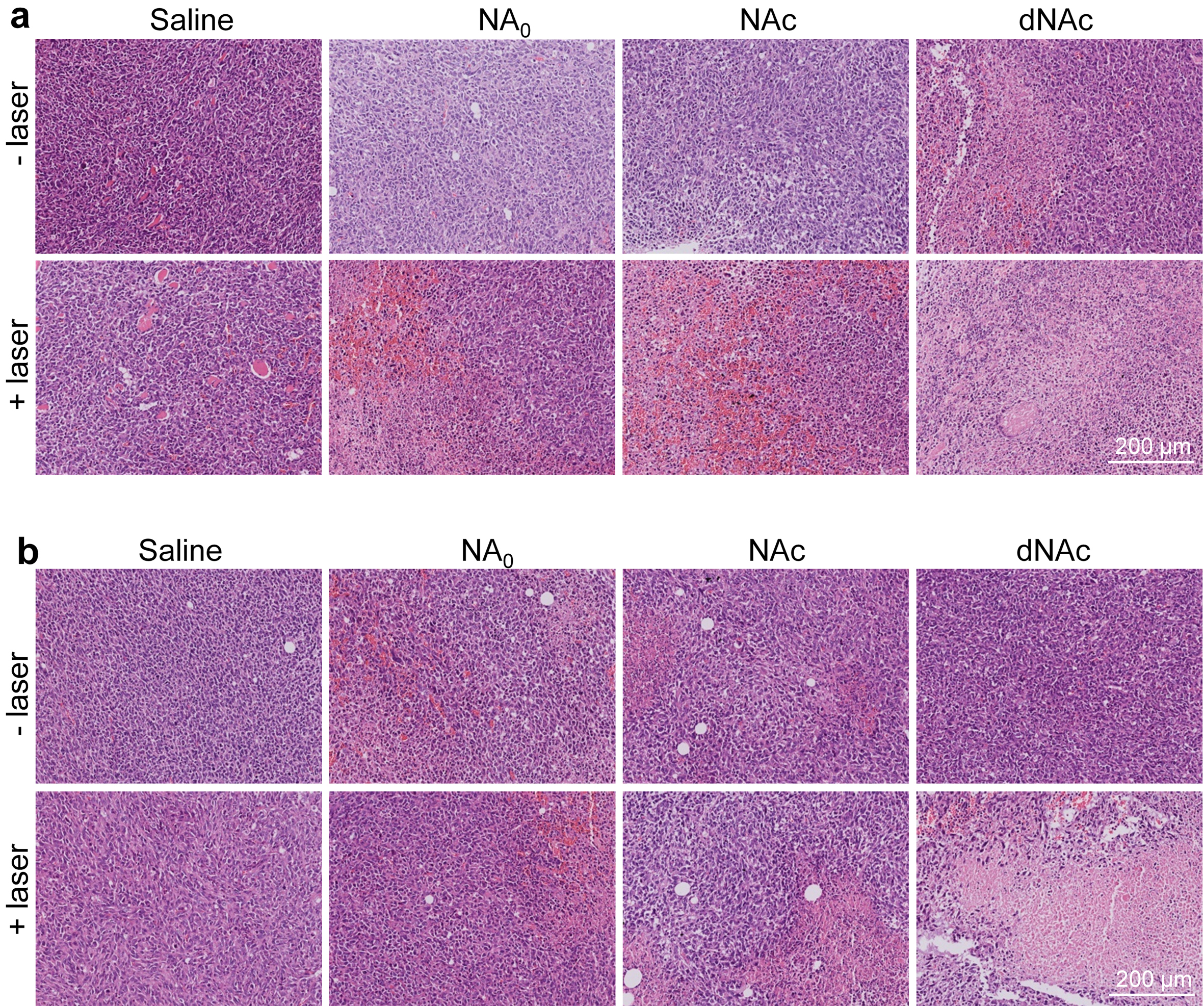


**Fig. S9.** H&E staining images of primary tumors (a) and distant tumors (b) from 4T1 tumor-bearing mice after intravenous injection of NA_0_, NAc and dNAc with or without NIR-II laser irradiation.


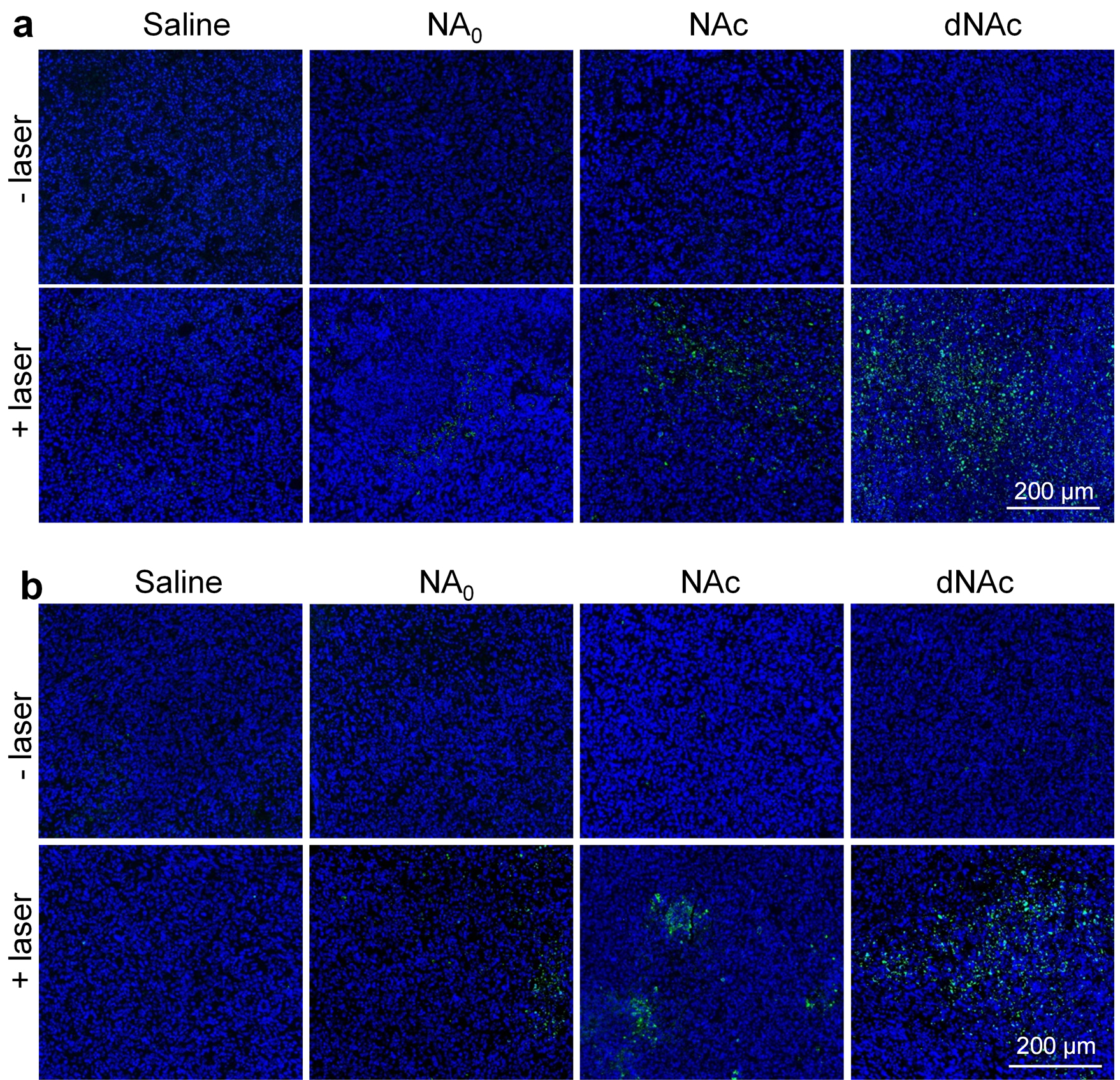


**Fig. S10.** TUNEL staining images of primary tumors (a) and distant tumors (b) from 4T1 tumor-bearing mice after intravenous injection of NA_0_, NAc and dNAc with or without NIR-II laser irradiation.


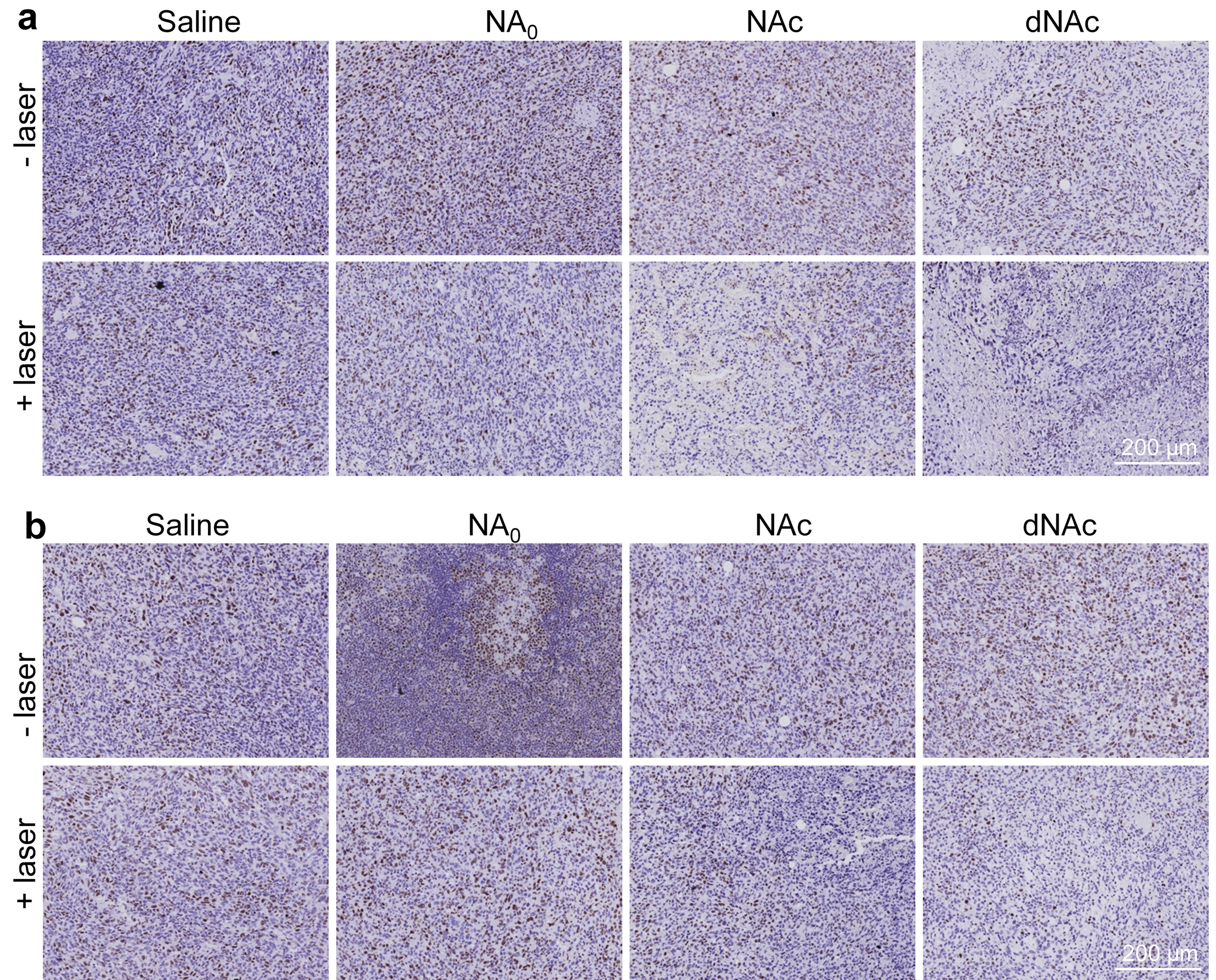


**Fig. S11.** Ki67 staining images of primary tumors (a) and distant tumors (b) from 4T1 tumor-bearing mice after intravenous injection of NA_0_, NAc and dNAc with or without NIR-II laser irradiation.


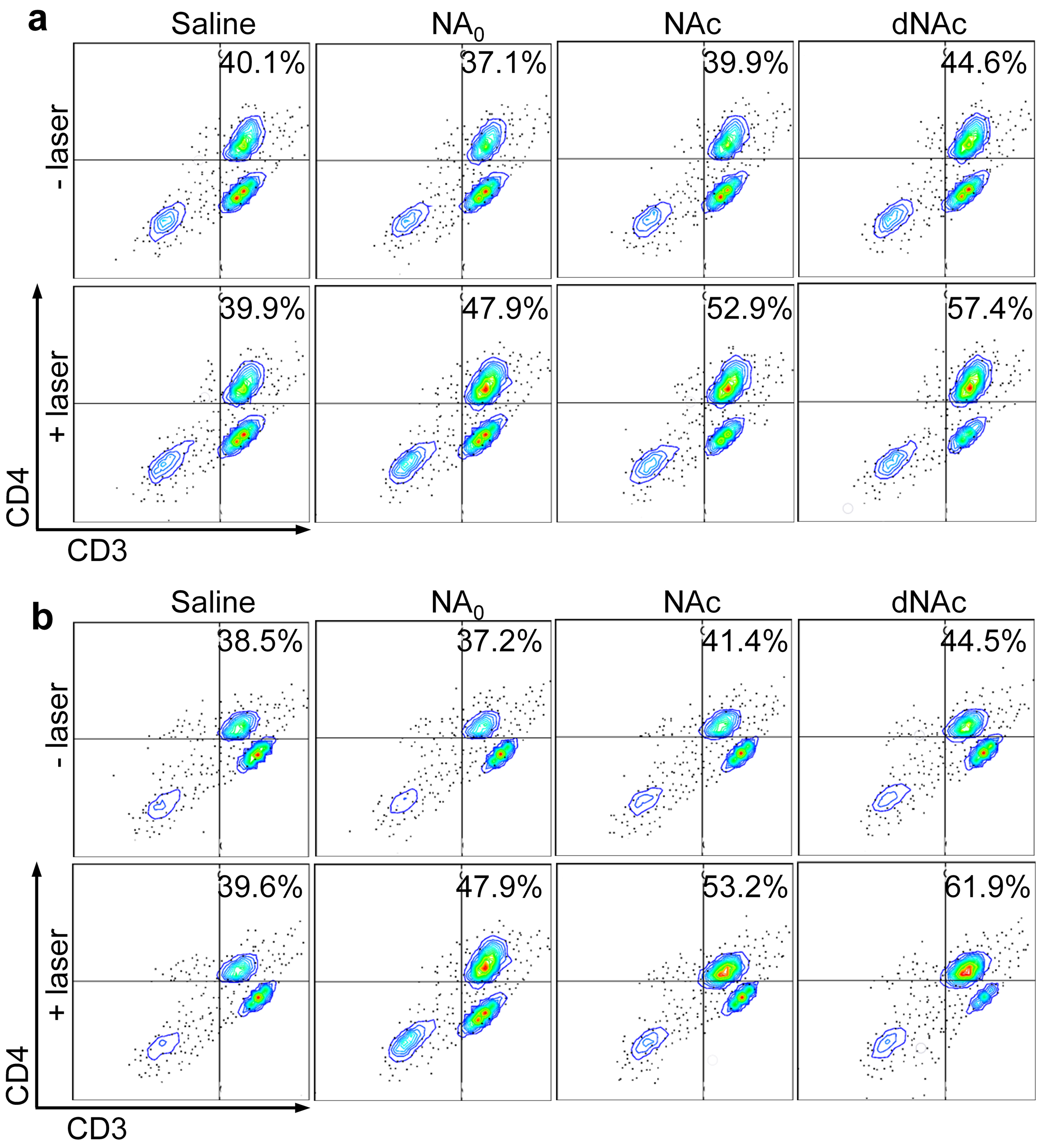


**Fig. S12.** Flow cytometry assay of CD3^+^CD4^+^ T cells in primary tumors (a) and distant tumors (b) of mice after intravenous injection of nanoagonists with or without NIR-II laser irradiation.


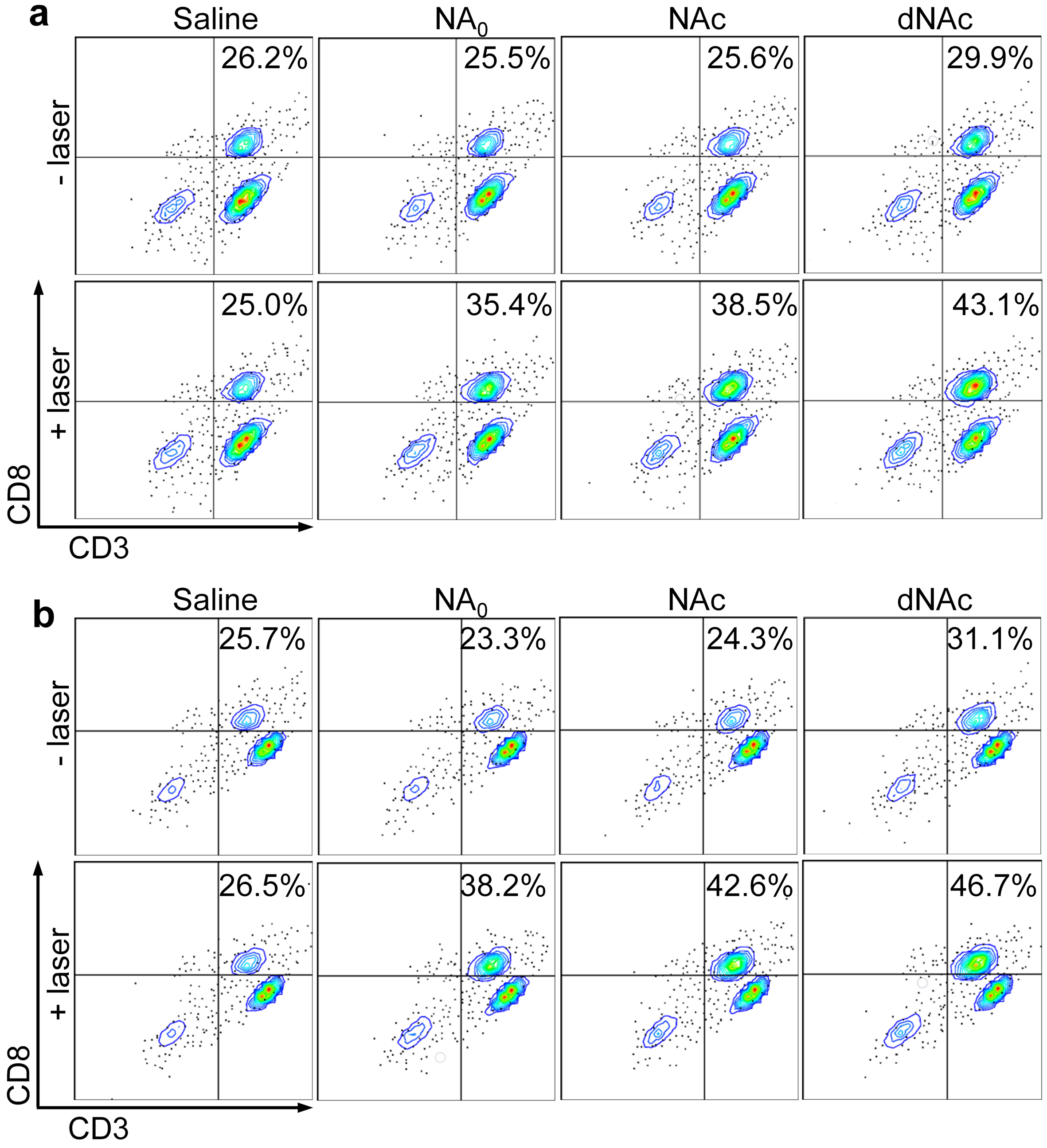


**Fig. S13.** Flow cytometry assay of CD3^+^CD8^+^ T cells in primary tumors (a) and distant tumors (b) of mice after intravenous injection of nanoagonists with or without NIR-II laser irradiation.


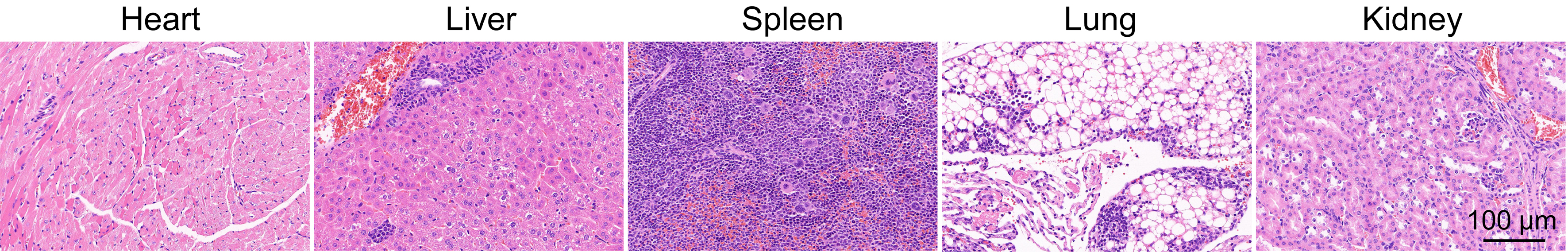


**Fig. S14.** H&E staining images of heart, liver, spleen, lung, and kidney of mice after intravenous injection of BSA-FeS_2_ nanoparticles.


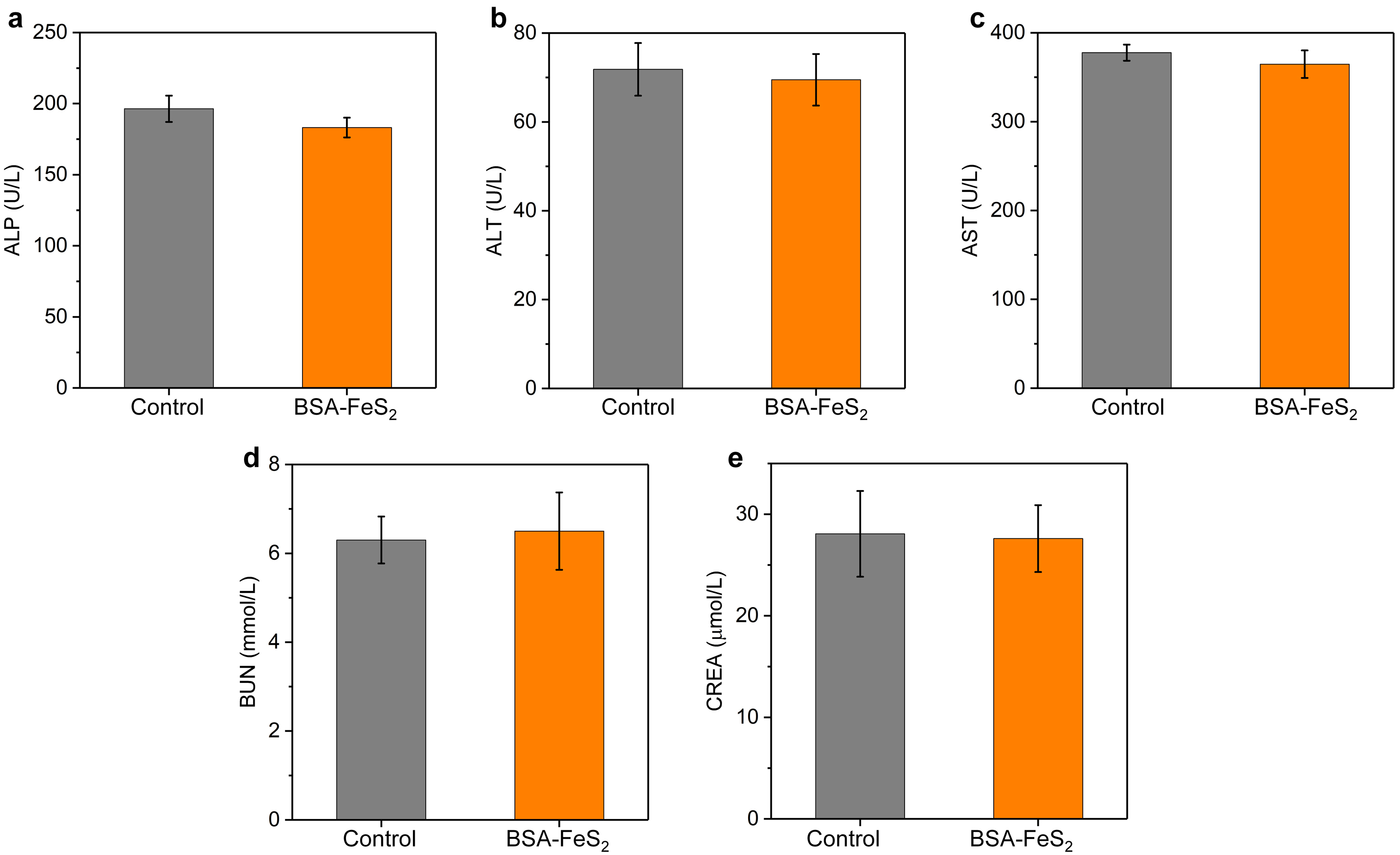


**Fig. S15.** The serum levels of (a) alkaline phosphatase (ALP), (b) alanine aminotransferase (ALT), (c) aspartate aminotransferase (AST), (d) blood urea nitrogen (BUN) and (e) creatinine (CREA) in mice after intravenous injection of BSA-FeS_2_ nanoparticles.


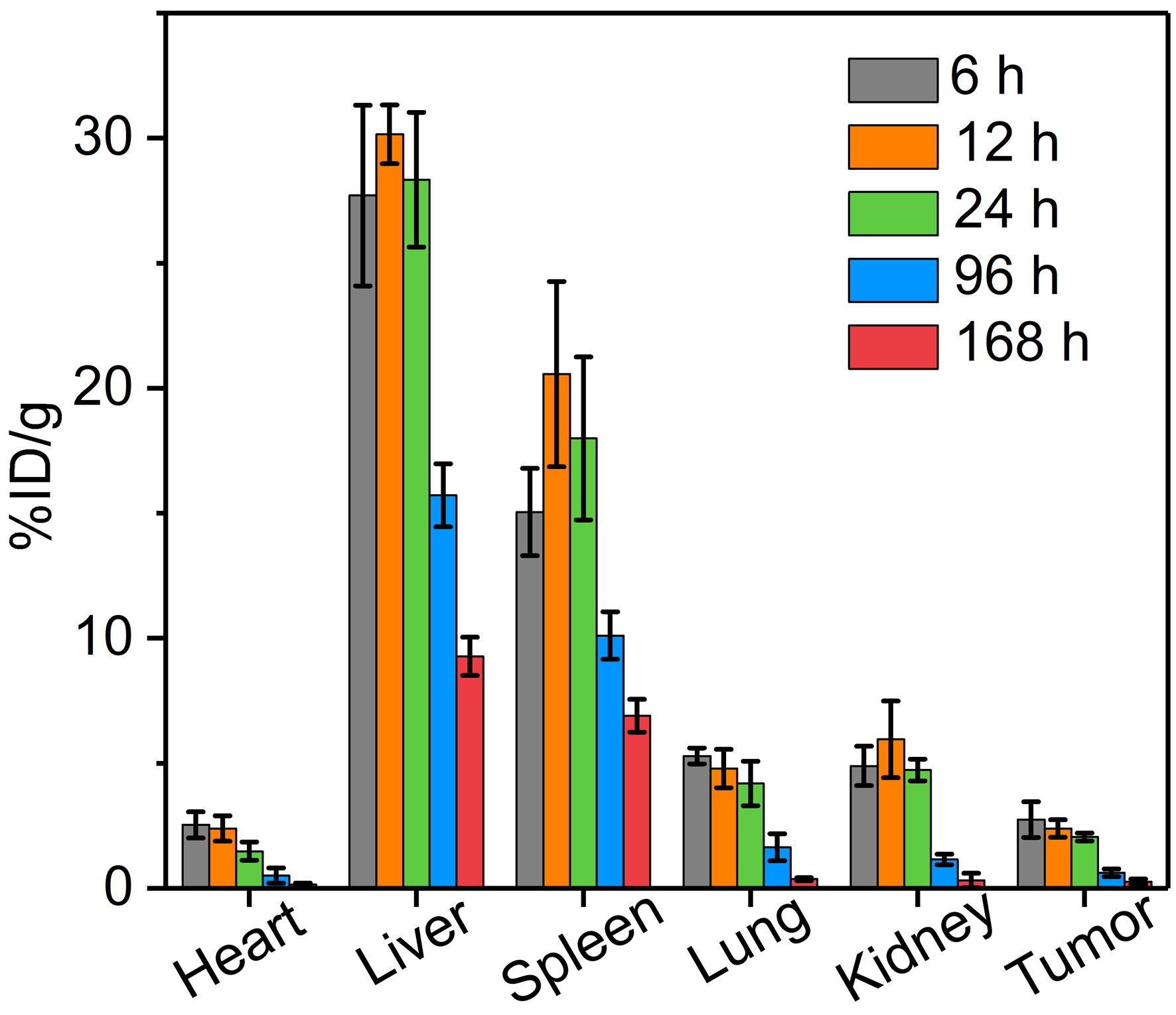


**Fig. S16.** Biodistribution of BSA-FeS_2_ nanoparticles in heart, liver, spleen, lung, kidney, and tumors at different post-injection timepoints.


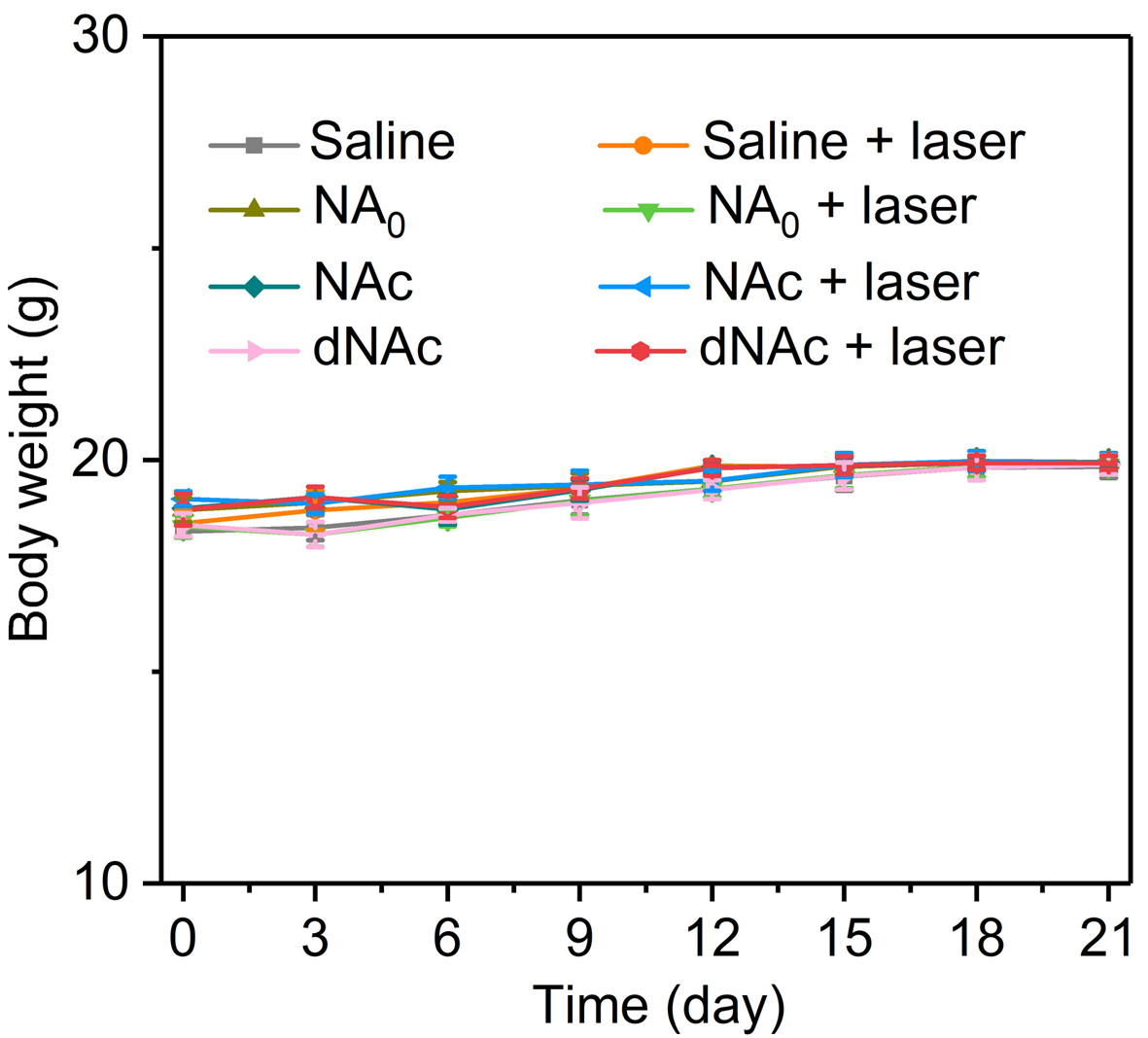


**Fig. S17.** Body weight of 4T1 tumor-bearing mice after intravenous injection of NA_0_, NAc or dNAc with or without NIR-II laser irradiation for different days.


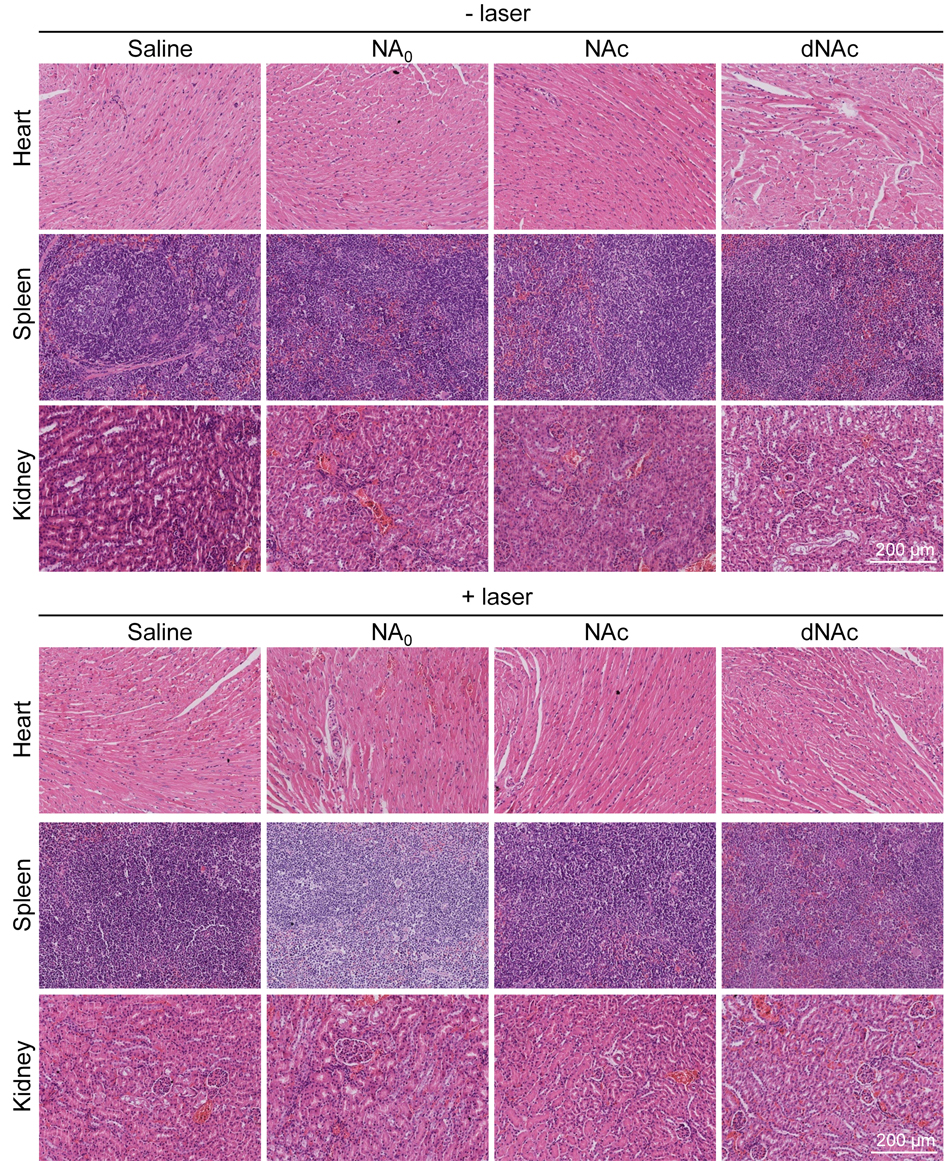


**Fig. S18.** H&E staining images of heart, spleen, and kidney of mice after different treatments for 21 days.


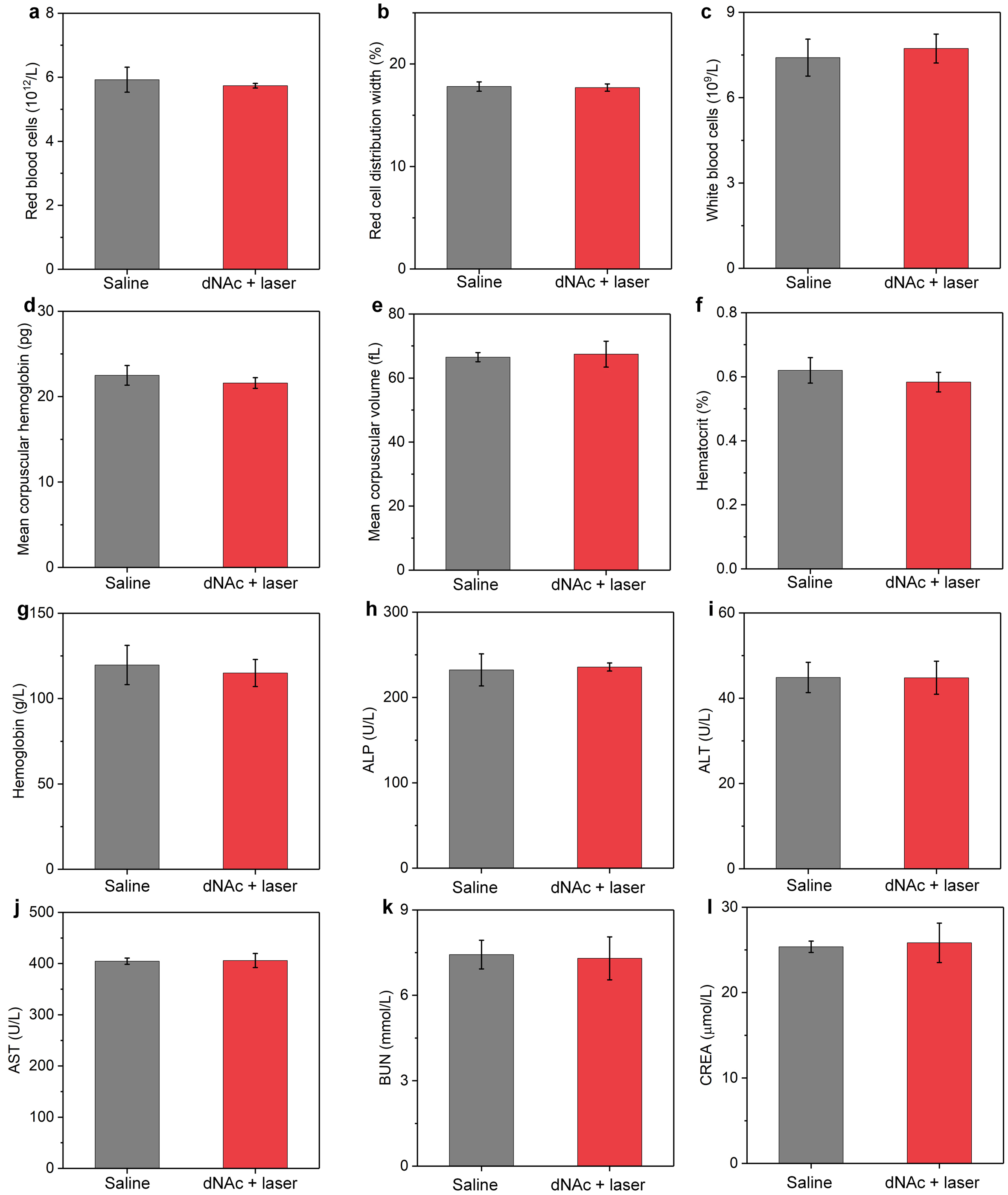


**Fig. S19.** The level of (a) red blood cells, (b) red cell distribution width, (c) white blood cells, (d) mean corpuscular hemoglobin, (e) mean corpuscular volume, (f) hematocrit, (g) hemoglobin, (h) ALP, (i) ALT, (j) AST, (k) BUN and (l) CREA in blood samples of control mice and mice after dNAc-mediated therapy.
